# Molecular pharmacodynamics of amoxicillin-clavulanic acid for urinary tract infections caused by *Escherichia coli*

**DOI:** 10.1038/s41467-026-74323-2

**Published:** 2026-06-13

**Authors:** Vineet Dubey, Christopher Darlow, Alessandro Gerada, Jennifer Unsworth, Esha Sheth, Nada Reza, Nicola Farrington, Sam Haldenby, Daniel Warren, Xuan Liu, Alexander Howard, William Hope

**Affiliations:** 1 Department of Clinical Pharmacology and Therapeutics, University of Liverpool, Liverpool, United Kingdom; 2 Liverpool Clinical Laboratories, University Hospitals of Liverpool Group, Liverpool, United Kingdom; 3 Centre for Genomic Research, University of Liverpool, Liverpool, United Kingdom

**Keywords:** Bacterial infection, Molecular biology

## Abstract

Amoxicillin-clavulanic acid (AMX-CLV) is a widely used oral β-lactam/β-lactamase inhibitor combination against *Escherichia coli*. Clinical success is largely confined to urinary tract infections. The mechanistic basis for this site-specific efficacy remains unclear. Using a hollow-fibre infection model to replicate human plasma and urinary pharmacokinetics, we show that plasma-like exposures rapidly select for pre-existing resistant subpopulations; whereas, urinary exposures produce sustained bactericidal activity without resistance emergence. Genomic and transcriptomic analyses following plasma drug exposure reveal that treatment selectively enriches pre-existing resistant lineages already harbouring oxidative-stress-associated mutations that activate the SOS response and drive IS-mediated amplification of *blaTEM-1*, leading to β-lactamase hyperproduction and treatment failure. In contrast, the high urinary concentrations of clavulanic acid exert direct antibacterial activity, eradicating these subpopulations. Our findings demonstrate that local pharmacokinetic environments fundamentally shape evolutionary trajectories under β-lactam/β-lactamase inhibitor therapy, explaining the restricted efficacy of AMX-CLV and revealing a dynamic interplay between stress responses, genome plasticity, and drug partitioning that governs treatment outcome.

## Abstract (web summary)

Using pharmacodynamic, genomic, and transcriptomic analyses, authors show that amoxicillin clavulanic acid selectively eradicates TEM-1-producing *Escherichia coli* in urinary tract infections by exploiting high urinary clavulanate exposure.

## Introduction

E*scherichia coli*, a leading cause of community and hospital-acquired infections, can express many antibiotic resistance mechanisms^1,2^. *E. coli* is often resistant to narrow-spectrum penicillins such as amoxicillin, and this is mediated by Ambler Class A β-lactamases, such as SHV and TEM enzymes^3,4^. These enzymes are inhibited by β-lactamase inhibitors such as clavulanic acid and tazobactam. Hence, combinations of antibiotics such as amoxicillin-clavulanic acid (AMX-CLV) and piperacillin-tazobactam are extensively used as first-line agents^5–7^. Since most *E. coli* disease occurs in ambulatory settings, orally bioavailable agents such as AMX-CLV are strategically valuable. Hence, a deep understanding of the pharmacodynamics and resistance liabilities of AMX-CLV is paramount.

Emerging evidence suggests that clinical success with AMX-CLV against *E. coli* is largely confined to urinary tract infections. The European Committee on Antimicrobial Susceptibility Testing (EUCAST) currently restricts recommendations for oral AMX-CLV to the treatment of UTIs^8^. Understanding the mechanistic basis for site-specific AMX-CLV efficacy could enable novel treatment strategies, including more informed, compartment-specific use of existing β-lactam/β-lactamase inhibitor combinations rather than the development of new antimicrobial agents. Such strategies may: (i) preserve a clinically useful and widely available oral agent; (ii) reduce treatment failures and associated morbidity and mortality; and (iii) help curb the global AMR crisis.

Here, we provide an understanding that when AMX-CLV is used for *E. coli*, its use should be restricted to urinary tract infections (UTI). We used a hollow-fibre infection model (HFIM) to replicate human-like pharmacokinetics of AMX-CLV against TEM-1 β-lactamase-producing *E. coli*. Although initial killing was achieved, AMX-CLV exposure enabled the expansion of pre-existing resistant subpopulations. Genomic analyses identified numerous mutational pathways that enhanced oxidative stress, activation of the SOS response, and mobilised *blaTEM-1* via Insertion Sequences (IS)-mediated translocatable units, increasing gene copy number and driving β-lactamase hyperproduction. Simulation of plasma and urinary profiles revealed that site-specific drug exposure determines whether AMX-CLV can suppress these resistant lineages, providing a molecular and pharmacological explanation for its limited efficacy outside the urinary tract.

## Results

### Clinical *E. coli* isolates exhibit variable β-lactam susceptibility despite shared AMX-CLV MICs

All 30 isolates had MICs >64 mg/L to AMX alone. However, the combination of AMX-CLV (2 mg/L of CLV) resulted in a 2- to 16-fold reduction in MICs (Table 1, see supplementary Table S1). A total of 16/30 isolates had MIC values ≤ 8 mg/L, which is the current AMX-CLV susceptibility breakpoint for Enterobacterales in all contexts except uncomplicated UTIs^9^. From the 30 clinical *E. coli*, B50, K35 and K67 were chosen for HFIM work as all three had an AMX-CLV MIC of 4 mg/L (Table 1), providing a matched susceptibility baseline. These strains carried TEM-1 and SHV-1 β-lactamases and originated from distinct clinical backgrounds.

**Table 1:** Minimum inhibitory concentration of AMX and AMX-CLV combination and AMR genes in UTI *E. coli* clinical isolates

Strain ID	Source	MIC (mg/L)		Resistance genes
		AMX	AMX-CLV	
B50	Blood	>64	4	**Efflux pumps:** *mdtFGH, acrAB-tolC, emrAB*, **β-lactamase:** *tem-1, EC-5* **Aminoglycoside inactivation:** *aadA, aph(6)-Id* **Other:** *arnT, pmrF, eptA, dfyA, sul1, sul2, tetB*
K35	Blood	>64	4	**Efflux pumps:** *acrAB-tolC* **β-lactamase:** *tem-1, EC-5* **Aminoglycoside inactivation:** *aph(3”)-Ib, aph(6)-Id* **Other:***sul2, arnT*
K67	Tissue	>64	4	**Efflux pumps:*** acrAC-tolC, mdtABC, acrEF*, **β-lactamase:** *shv-1, EC-5* **Aminoglycoside inactivation:** *aadA* **Other:***eptA, arnT*

### Rapid emergence of resistant subpopulations under plasma-like AMX-CLV exposure

*E. coli* isolates were exposed to the following human-like plasma profiles in the HFIM for 48 h: untreated control, AMX-CLV 500/125 mg q8h, and AMX-CLV 1000/125 mg q8h.

**Fig. 1:**
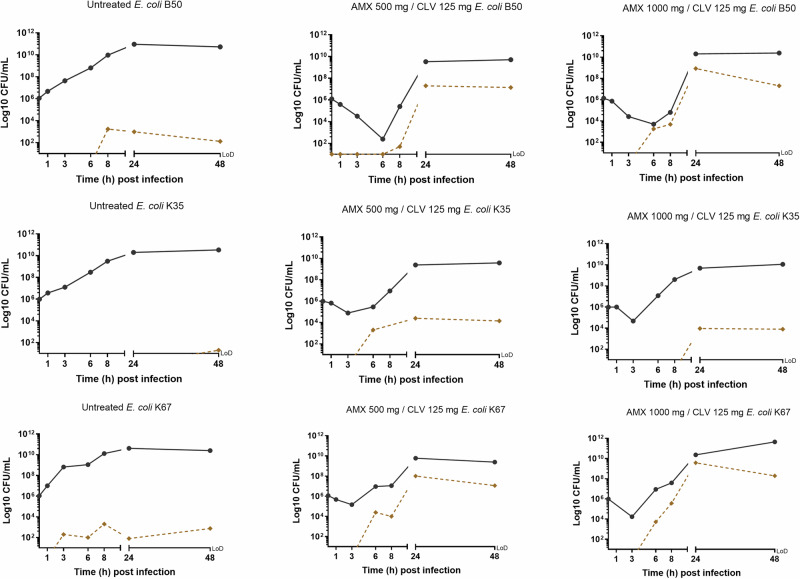
Plasma pharmacokinetic simulations reveal rapid resistance emergence in TEM-1 producing *E. coli* isolates. HFIM experiments simulating human plasma pharmacokinetics of AMX and CLV. Black lines indicate total bacterial counts (log₁₀ CFU /mL); orange dashed lines represent resistant subpopulations detected on AMX-CLV-supplemented plates. **Top row:** *E. coli* B50 exposed to AMX-CLV at 500 mg and 125 mg or 1000 mg and 125 mg shows an initial bactericidal effect followed by regrowth within 24–48 h, consistent with selection of resistant subpopulations. **Middle row:** *E. coli* K35 exhibited similar kinetics, with transient killing followed by recovery of resistant colonies despite dose escalation. **Bottom row:** *E. coli* K67 displayed early growth suppression followed by rapid rebound, confirming that treatment failure arises across distinct TEM-1/SHV-1 expressing lineages. Drugs were administered every 8 h throughout the duration of the experiment. Pharmacokinetic sampling was performed on Day 1 and on the day prior to experiment completion.

The untreated arm exhibited logarithmic growth, with few resistant colonies recovered on drug-containing plates. Following drug treatment, initial bacterial killing was observed. However, there was rapid regrowth and emergence of a resistant sub-population (Fig. 1). Resistance was confirmed by plating samples on MH agar containing AMX/CLV (32/2.5 mg/L). Colonies isolated from drug-containing plates displayed MIC values in the range of 32–128 mg/L; whereas, colonies from drug-free plates in the untreated arm retained their original MIC values. The corresponding drug exposure profiles for each arm are shown in Supplementary Fig. 1. We observed differences in the AMX profiles among the treatment groups, with AMX lower than the desired peak concentration at 72–80 h (see Supplementary Fig. 1). This may have been attributed to increased TEM-1 in the hollow-fibre cartridges in 2–3 days, leading to AMX hydrolysis.

### Pharmacodynamic failure arises from rare, pre-existing TEM-1 overexpressing *E. coli* lineages

**Fig. 2:**
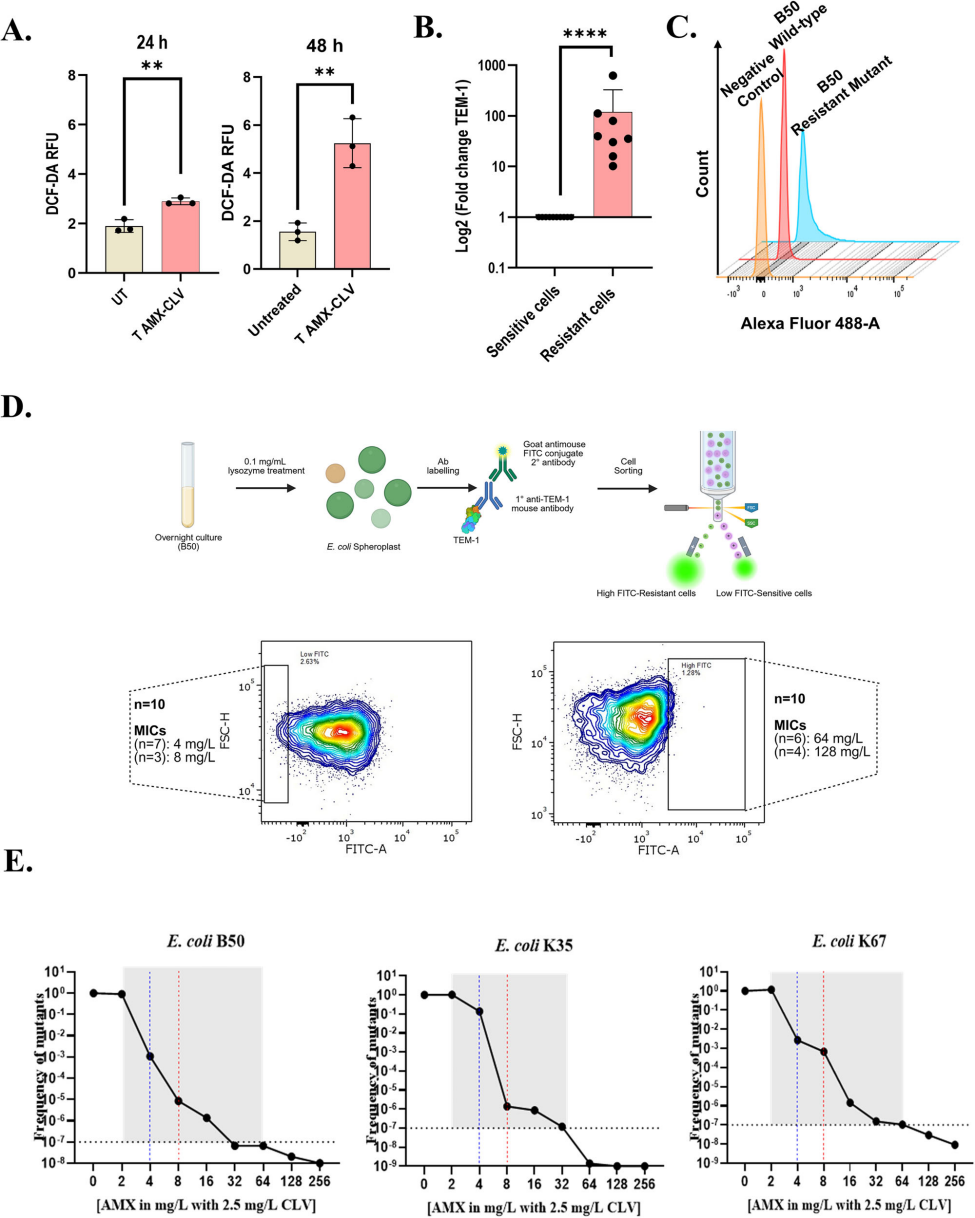
Pre-existing resistant subpopulations with high *blaTEM-1* expression drive treatment failure under AMX-CLV exposure. **A** Intracellular reactive oxygen species (ROS) levels in *E. coli* B50 increased significantly after exposure to plasma PK profiles of AMX–CLV (500 mg + 125 mg) for 24 h and 48 h compared with untreated controls (UT). ROS levels were measured by DCF-DA fluorescence. Data are presented as mean ± s.d.; each point represents an independent biological replicate (*n* = 3 independent samples per time point). Biological replicates correspond to independently isolated samples recovered at each time point from the HFIM. Each biological replicate consisted of two technical replicate measurements, which were averaged prior to analysis. Statistical comparisons were performed using a two-tailed unpaired Student’s *t*-test. For comparison, *t* = 5.959, d.f. = 4, *P* = 0.0040 (95% CI = 0.534–1.465). No adjustment for multiple comparisons was applied. **B** RT-qPCR analysis confirmed that resistant cells expressed blaTEM-1 at higher levels than sensitive cells. Data are presented as medians with interquartile ranges; each point represents an independent biological replicate (*n* = 8 independent cultures per group). Statistical comparisons were performed using a two-tailed Mann-Whitney *U*-test (*U* = 0, *P* < 0.0001). No adjustment for multiple comparisons was applied. **C** TUNEL assay detects DNA double-strand break signal in AMX-CLV-selected resistant subpopulations. Sensitive and resistant *E. coli* B50 cells were quantified by flow cytometry. **D** Flow cytometry of antibody-labelled spheroplasts demonstrated two distinct subpopulations with high or low TEM-1 abundance, and subsequent cell sorting showed that low-FITC cells exhibited MICs of 4–8 mg/L, whereas high-FITC cells exhibited MICs of 64–128 mg/L. *Created in BioRender. Farrrington, N. (2026)* https://BioRender.com/ipn2ruc. **E** Population analysis profiles of *E. coli* B50, K35, and K67 further revealed heterogeneous resistance, with resistant fractions persisting across a wide range of AMX concentrations (0.5–256 mg/L) in the presence of a fixed CLV concentration (2.5 mg/L), consistent with the selection of pre-existing resistant subpopulations that ultimately undermine treatment efficacy. Blue and red dashed lines are MIC for strain and EUCAST breakpoint for AMX-CLV, respectively.

β-lactams inhibit penicillin-binding proteins involved in cell wall synthesis, and the resulting cell wall damage can trigger downstream stress responses, including the generation of reactive oxygen species (ROS)^10^. To examine ROS dynamics during clinically relevant plasma exposures of AMX-CLV, we sampled *E. coli* B50 in a HFIM at 24 h and 48 h with arms consisting of an untreated control and a concentration time profile of AMX/CLV corresponding to 500/125 mg q8h. Total bacterial densities were comparable over time, but treated populations became increasingly dominated by colonies growing on AMX-CLV plates. These colonies exhibited significantly higher intracellular ROS than untreated controls (mean ± s.d.; *n* = 6; *P* < 0.01, Student’s *t*-test) (Fig. 2A), indicating that the drug-selected fraction experienced elevated oxidative stress even after net killing had plateaued.

To test whether ROS directly contributed to resistance generation, we serially passaged B50 in escalating AMX-CLV concentrations (8–32 mg/L) with or without the ROS quencher thiourea (100 mM). The frequency of resistance between these experimental conditions was indistinguishable (see Supplementary Fig. 2), suggesting there was no de novo ROS-associated mutagenesis. Together with modest increases in double-strand breaks detected by TUNEL assays (Fig. 2C), these data suggest that elevated ROS is a correlate of a selected state rather than a primary driver of new mutational events that contribute to AMX-CLV resistance.

We next investigated whether there were pre-existing resistant subpopulation(s) that preceded drug exposure. Flow cytometry using anti-TEM-1-FITC antibodies revealed basal heterogeneity in TEM-1 abundance in drug-unexposed B50 cultures. Cells sorted from the lowest-fluorescence gate (representing ~97–99% of the total population) exhibited AMX-CLV MICs of 4–8 mg/L, whereas those from the highest-fluorescence gate ( ~ 1–3%) displayed MICs of 64–128 mg/L (Fig. 2D). This demonstrated phenotypically distinct subpopulations with elevated TEM-1 expression are present prior to antibiotic exposure. Population-analysis profiling (PAP) provided consistent results, with clinical isolates (B50, K35, K67) each showing resistant subfractions spanning AMX-CLV MICs of 8–128 mg/L (Fig. 2E). These frequencies ( ~ 10^−3^-10^−4^) are sufficient to account for the bacterial regrowth in HFIM experiments.

Colonies recovered from drug-containing plates displayed striking *blaTEM-1* upregulation ( ~ 8- to >600-fold relative to ancestral cells) and modest evidence of DNA damage (Fig. 2A, C). Combined with flow cytometry and PAP data, these findings support a scenario in which plasma-relevant AMX-CLV exposures preferentially enrich pre-existing TEM-1-high lineages rather than drive the de novo emergence of resistance. The in vitro pharmacodynamic data identify the expansion of a TEM-1 overexpressing subpopulation as a key determinant of regrowth following systemic AMX-CLV exposure.

### Resistant subpopulations display transcriptional rewiring and oxidative stress-linked genomic instability

To investigate the molecular basis of resistance in the AMX-CLV selected subpopulations, we compared the transcriptomes and genomes of resistant-versus-sensitive isolates derived from the same parental strains.

**Fig. 3:**
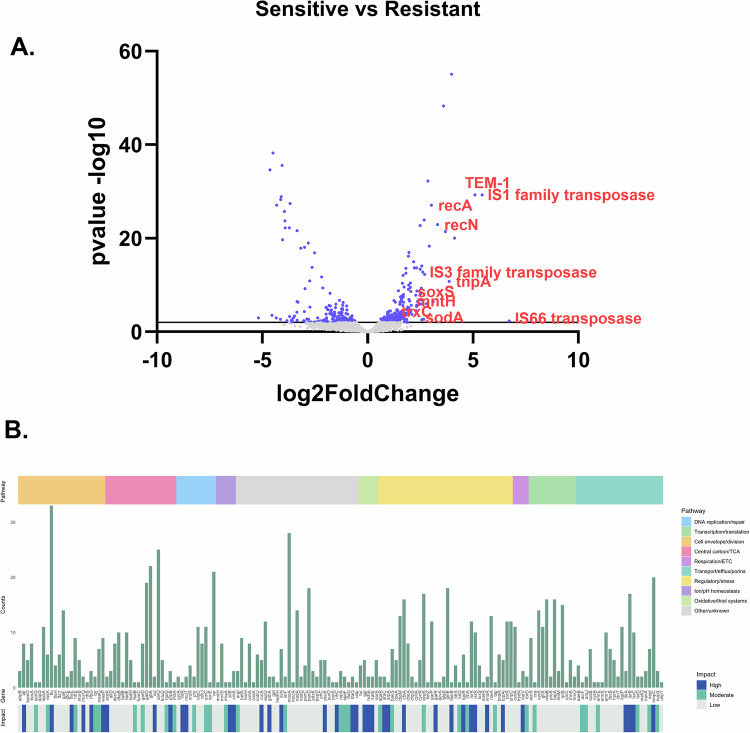
Transcriptomic and genomic alterations distinguish resistant from sensitive subpopulations. **A** Differential RNA-seq analysis of resistant versus sensitive *E. coli* subpopulations revealed upregulation of stress- and mobility-associated genes (e.g., *blaTEM-1*, *recA*, IS-family transposases) and downregulation of core metabolic pathways. RNA-seq was performed with three biological replicates (*n* = 3). Differential expression analysis was conducted using DESeq2 with negative binomial modelling, library size normalisation, and dispersion estimation. Low-abundance genes were filtered prior to analysis. Wald tests were used for statistical inference, with p-values adjusted using the Benjamini-Hochberg FDR method. **B** Whole-genome sequencing of resistant isolates identified high-impact mutations such as stop codon loss and frameshifts, moderate-impact nonsynonymous SNPs, and low-impact upstream changes, distributed across genes involved in DNA repair, oxidative stress defence, metabolism, and regulatory functions. Visualisation of mutation frequency across functional categories shows resistant isolates are enriched for mutations in stress adaptation and genome plasticity pathways. Count represents how many unique variant sites we found in that gene that are present in the resistant mutants and absent in the ancestral isolates.

Differential RNA-seq analysis revealed extensive transcriptional remodelling in resistant populations (Fig. 3A). Genes associated with β-lactam resistance (*blaTEM-1*), DNA-damage repair (*recA, recN*), and multiple IS-family transposases were among the most strongly upregulated. These patterns indicate that the high-level *blaTEM-1* expression observed phenotypically is accompanied by activation of stress-response and mobile-element pathways, suggesting resistant subpopulations are transcriptionally primed to tolerate and adapt to AMX/CLV exposure.

Functional enrichment analysis of the differentially expressed genes revealed pathway-level reprogramming (See Supplementary Fig. 3). Resistant isolates upregulated core metabolic and translational processes (i.e., glycolysis, amino-sugar metabolism, ribosome biogenesis) while downregulating anabolic pathways such as amino-acid and cofactor biosynthesis. This shift implies metabolic prioritisation toward energy production and protein synthesis at the expense of long-term biosynthetic investment, a pattern typical of stress-adapted states that favour short-term survival under antibiotic pressure.

Whole-genome sequencing identified diverse mutations across functional categories (Fig. 3B). High-impact variants included frameshifts and premature stop codons; whereas, moderate-impact variants were largely nonsynonymous SNPs predicted to alter protein structure. Several mutations mapped to oxidative-stress and DNA-damage response genes (*mutT, katE, mntH, ahpC, gor*) and regulators of general stress responses (*rpoS, uspA*). These changes collectively point to chronic activation of oxidative-stress management and repair pathways in resistant isolates.

Notably, the occurrence of oxidative stress-associated mutations alongside transcriptional upregulation of IS-family transposases across independently evolved resistant isolates indicates coordinated stress and mobile-element activity in these populations. Increased transposase activity under DNA-stress conditions could facilitate rearrangements and gene amplifications, including expansion of *blaTEM-1* copy number.

### Increasing AMX-CLV exposure delays but cannot prevent resistance expansion

To evaluate how drug exposure profiles influence bacterial killing and resistance emergence, we used the HFIM to simulate the human pharmacokinetics of AMX and CLV against the TEM-1-producing *E. coli* strain B50. The two drug components were administered independently, allowing precise control of concentration-time profiles that cannot be achieved in vivo.

**Fig. 4:**
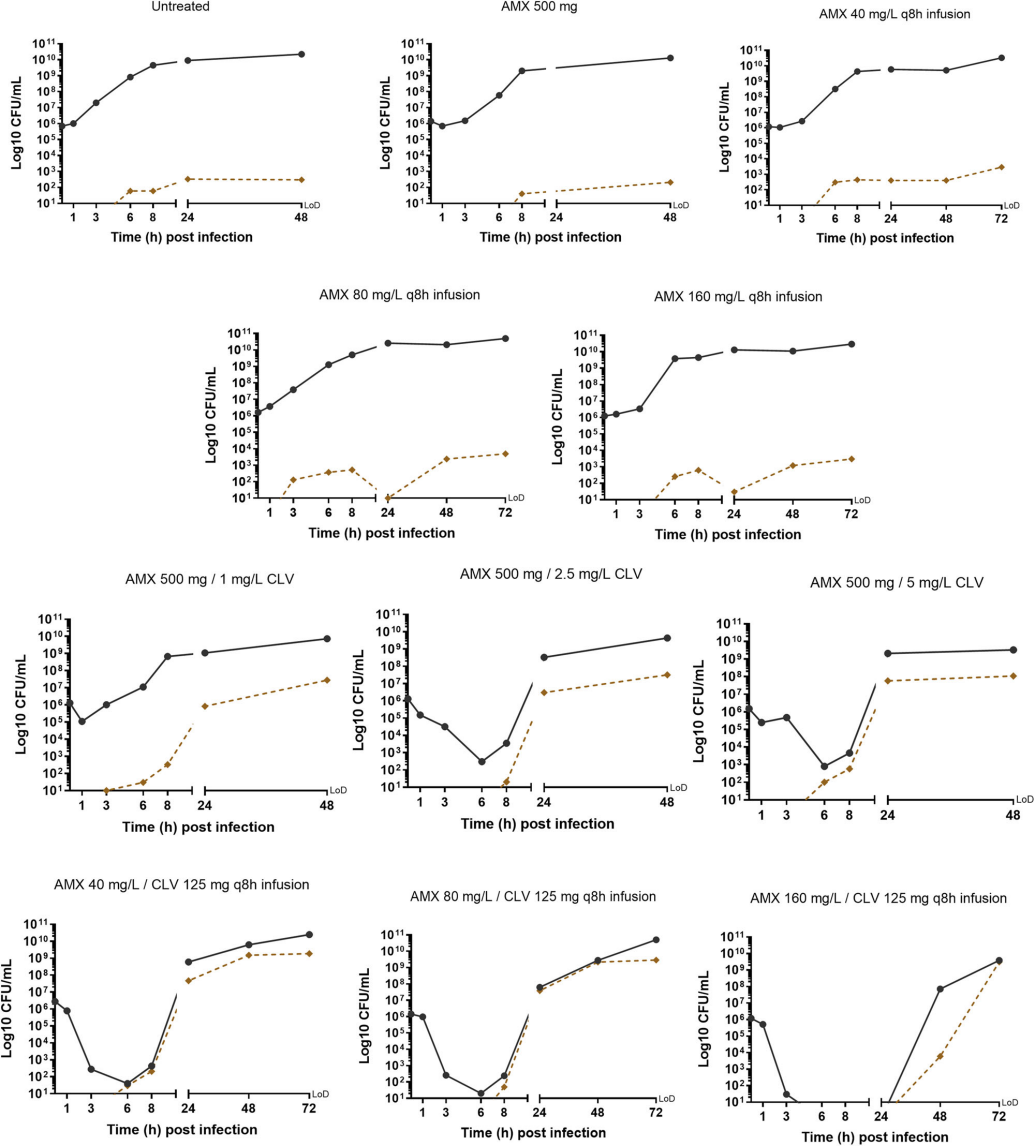
Increasing AMX-CLV exposure delays but does not prevent resistance expansion. HFIM experiments simulating human plasma pharmacokinetics of AMX and CLV against *E. coli* strain B50 producing TEM-1 β-lactamase. Each plot shows bacterial population dynamics (log_10_ CFU/mL) over time under varying doses and infusion regimens. Black lines denote total bacterial counts; orange dashed lines indicate resistant subpopulations detected on AMX-CLV-supplemented plates. **Top row:** Untreated control, AMX monotherapy (500 mg) and infusion of AMX 40 mg/L show uncontrolled bacterial growth. **Second row:** Infusion of AMX 80 and 160 mg/L shows uncontrolled bacterial growth. **Third row:** Increasing CLV concentrations (1–5 mg/L) with fixed AMX exposure initially enhanced killing but failed to prevent regrowth by 48 h, indicating that higher CLV exposure alone is insufficient to suppress resistance. **Bottom row:** Combined AMX-CLV regimens (40–160 mg/L AMX; 125 mg CLV) delayed but did not prevent re-emergence of resistant subpopulations, even at exposures exceeding clinically achievable plasma concentrations.

Plasma pharmacokinetics of AMX following oral dosing with 500 mg q8h and a continuous infusion of CLV to maintain flat concentrations between 1 and 5 mg/L were simulated. Increasing CLV exposure produced progressively faster and deeper initial killing, but in all arms bacterial regrowth occurred by 48 h, and resistant subpopulations ultimately dominated by 48 h (Fig. 4). Thus, higher CLV concentrations enhanced early bactericidal activity but were insufficient to prevent selection of resistant lineages.

Next, we varied AMX concentrations while fixing CLV exposure to simulate plasma pharmacokinetics corresponding to standard oral dosing (125 mg every 8 h). AMX was infused to maintain static free plasma concentrations of 40, 80, and 160 mg/L in the HFIM. Clinically relevant plasma concentrations refer to those achievable following oral AMX-CLV administration, for which reported peak AMX plasma concentrations are substantially lower than 160 mg/L. Accordingly, the higher AMX concentrations used here exceed oral exposure and were included to probe resistance dynamics under elevated systemic drug pressure. Increasing AMX concentrations accelerated bacterial killing. Cultures at 40 and 80 mg/L displayed regrowth after 48 h; whereas, at 160 mg/L, counts fell below the limit of quantification for 48 h before rebounding at 72 h (Fig. 4). Even these exposures, which approach or exceed the upper boundary of plasma concentrations achievable in humans, did not result in bacterial eradication. The corresponding drug exposure profiles for each arm are shown in Supplementary Fig. 4. As the regrowth was associated with a TEM-1-enriched subpopulation, we hypothesised that accelerated β-lactam degradation impaired attainment of the intended AMX-CLV exposures; consistent with this, nitrocefin hydrolysis by cells sampled from each dosing arm showed markedly increased β-lactamase activity compared with untreated controls (See Supplementary Fig. 5).

**Fig. 5:**
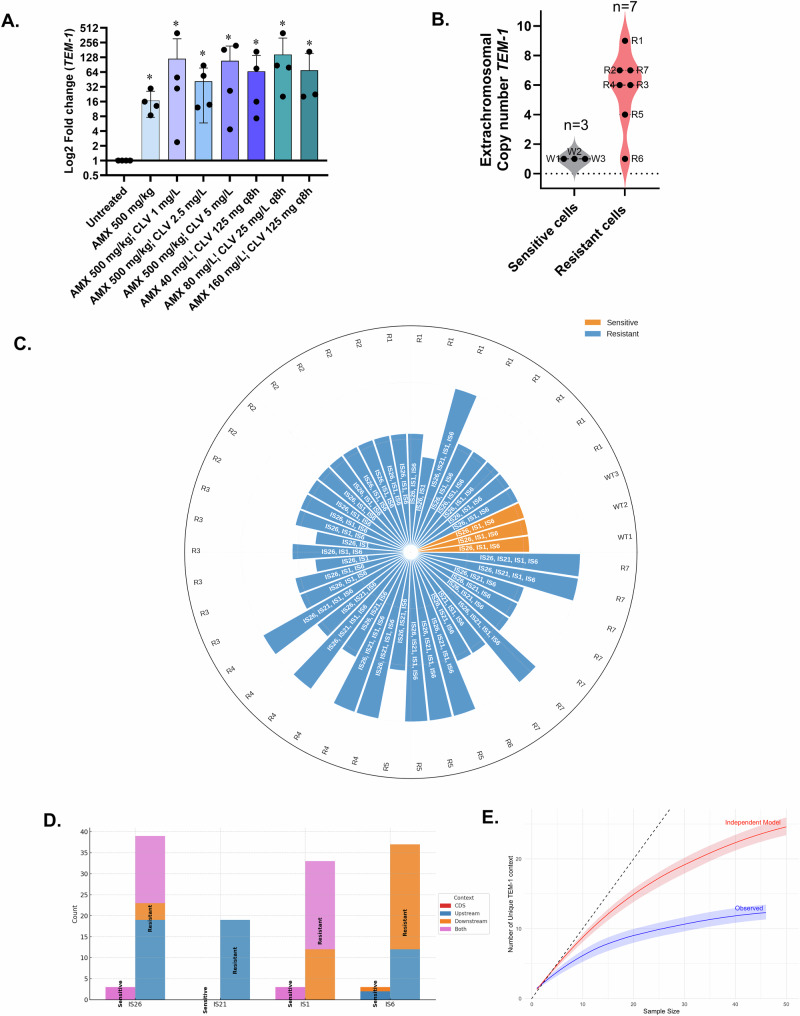
Amplification and structural remodelling of *blaTEM-1* in resistant subpopulations. **A** qPCR analysis of blaTEM-1 expression in resistant colonies recovered from dose-optimisation experiments shows elevated transcript levels compared with untreated controls. Data are presented as medians with interquartile ranges; each point represents an independent biological replicate. *n* = 4 independent colonies were analysed for untreated controls and all other treatment conditions. For the AMX (160 mg/L) and CLV (125 mg/L) treatment conditions, *n* = 3 independent biological replicates were used, which were sufficient to capture the observed variability for non-parametric analysis. Each biological replicate corresponds to a separately isolated colony processed independently. Technical replicates (qPCR reactions) were performed in triplicate and averaged prior to analysis. Statistical comparisons between each treatment and the untreated control were performed using a two-tailed Mann-Whitney *U*-test (*P* = 0.0286). Median values were 1.00 for untreated controls and 14.48 for AMX-treated samples, with a Hodges-Lehmann difference of 13.48. No adjustment for multiple comparisons was applied. **B** Quantification of extrachromosomal blaTEM-1 copy number in sensitive (*n* = 3) and resistant (*n* = 7) isolates, illustrating heterogeneity in copy number across isolates. Data are presented as individual biological replicates; no statistical test was performed as this panel is intended to provide a descriptive comparison of variability. **C** Circular mapping of the immediate genetic neighbourhood of *blaTEM-1* in sensitive (orange) and resistant (blue) isolates, highlighting diversification of insertion sequence (IS) boundaries around *blaTEM-1*. **D** Distribution of IS families and their genomic positions relative to coding sequences (CDS), upstream, downstream, or multiple contexts in sensitive and resistant isolates. **E** Rarefaction analysis comparing the observed diversity of *blaTEM-1* contexts (blue line) with a null model assuming independent IS family insertions (red line) and the theoretical upper bound (dashed line), showing that resistant isolates are constrained to a limited repertoire of TU architectures. Data are presented as median values (LOESS-smoothed), with shaded error bands representing the interquartile range (25th–75th percentiles).

**Fig. 6:**
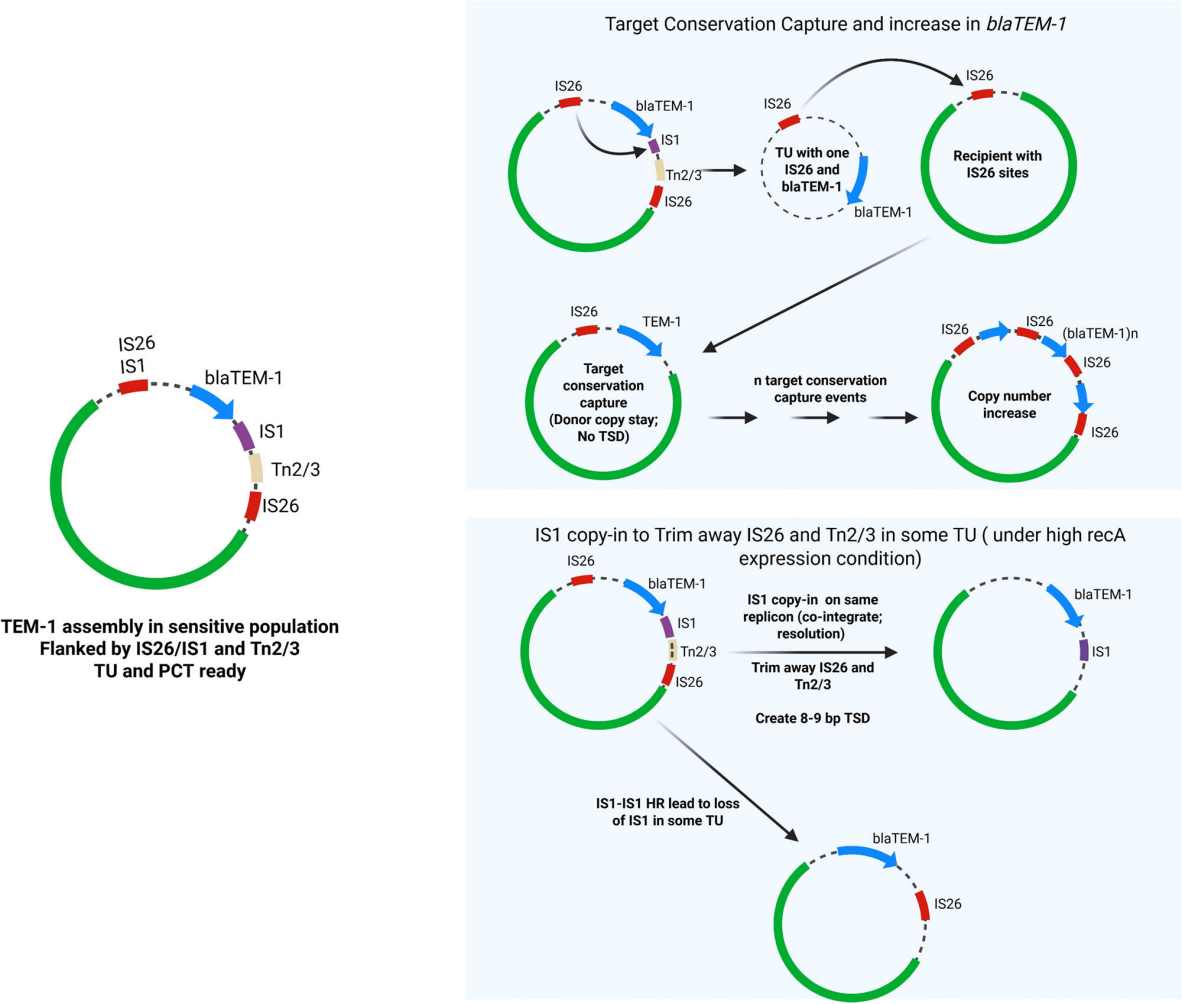
IS-mediated remodelling of *blaTEM-1* transposition units. In sensitive isolates, *blaTEM-1* is flanked by IS26/IS1 and Tn2/3. Under drug pressure, IS26-driven target conservation capture expands copy number, while IS1 insertions and recombination prune or replace TU boundaries. These processes generate the limited set of TU architectures observed in resistant isolates. *Created in BioRender. Farrrington, N. (2026)* https://BioRender.com/bofk746.

Collectively, these experiments show that dose escalation of either component confers only transient suppression of resistant subpopulations. While the precise molecular mechanisms were not delineated for each regimen, subsequent genomic analyses revealed convergent signatures of *blaTEM-1* amplification conditions (see Figs. 5, 6), suggesting a shared adaptive pathway to resistance.

### IS-mediated amplification and structural remodelling of *blaTEM-1* drive high-level resistance

To define the genomic basis of resistance emerging under intensified AMX-CLV exposure, we quantified *blaTEM-1* expression and copy number in isolates recovered from HFIM dose-modulation experiments. Quantitative RT-PCR showed that all resistant isolates expressed *blaTEM-1* at markedly higher levels than untreated controls (Fig. 5A). Expression increased by ~10- to >100-fold for all treatment groups when compared to untreated controls. Transcriptional upregulation of *blaTEM-1* is a consistent feature of resistant populations across regimens.

To test whether increased gene dosage contributed to this overexpression, we quantified extrachromosomal transposition units (TUs) carrying *blaTEM-1*. Resistant isolates contained 1 to 9 copies per cell, compared with the single-copy baseline in sensitive controls (Fig. 5B). Individual colonies exhibited heterogeneous amplification patterns, suggesting that gene-dosage effects arise through dynamic genomic rearrangements and the mobilisation of *blaTEM-1*-containing elements.

We next examined the local genetic environment of *blaTEM-1* in untreated (WT1-WT3) and resistant isolates (R1-R7). In sensitive strains, *blaTEM-1* resided within a simple IS26/IS1-flanked transposition unit consistent with a single-copy mobile element. Resistant isolates showed diversified architectures enriched for additional IS families (IS21, IS6), yielding multiple distinct endpoints (Fig. 5C). Rather than random insertion, this pattern is explained most parsimoniously by the selective enrichment of rare, pre-existing high-copy configurations that confer a competitive advantage during AMX-CLV exposure.

Mapping the genomic positions of IS elements relative to coding sequences and regulatory regions confirmed that resistant isolates exhibit broader IS activity across contexts (Fig. 5D). Upstream insertions were rare and did not create canonical promoter motifs, suggesting that transcriptional activation arises primarily from gene amplification rather than promoter engineering.

To assess whether the observed diversity of *blaTEM-1* architectures could occur by chance, we compared the empirical data with a random-insertion model (Fig. 5E). Simulated independent insertions predicted substantially higher structural diversity than observed experimentally. In contrast, real data plateaued at ~12 unique architectures; far below the theoretical maximum of 625 possible combinations, indicating that *blaTEM-1* mobility is constrained to a limited repertoire of recurrent, evolutionarily favoured configurations.

To integrate these diverse transposition unit architectures into a coherent framework, we next developed a mechanistic model of IS-mediated *blaTEM-1* remodelling (Fig. 6). Under antibiotic pressure, IS26-driven replicative transposition or homologous recombination can duplicate the IS26-*blaTEM-1*-IS21 module in tandem arrays, whereas secondary recombination events may delete or replace specific IS boundaries, producing variants flanked by IS21 alone, IS1 alone, or no ISs at all. These processes generate a continuum of related architectures that differ in structure but share the outcome of increased *blaTEM-1* dosage.

Together, these results reveal that amplification of *blaTEM-1* through structured IS-mediated rearrangements rather than random transposition. This constrained genomic plasticity likely stabilises high-level β-lactamase production and underpins the reproducible resistance phenotypes observed across dosing regimens.

### Urinary drug exposures achieve sustained bacterial clearance and prevent resistance emergence

Given the predominantly renal elimination of AMX-CLV, we simulated urinary pharmacokinetics for both drugs using a physiology-based pharmacokinetic (PBPK) model (See supplementary Fig. 6). Simulations assumed a micturition frequency of q8h and generated urine *C*max values of 290 mg/L for AMX and 40 mg/L for CLV, consistent with urinary concentrations achieved after standard oral dosing of 500/125 mg q8h^11,12^. Using these parameters, we performed HFIM experiments as follows: untreated control, AMX monotherapy, CLV monotherapy, and AMX-CLV combination therapy. The corresponding drug exposure profiles for each arm are shown in Supplementary Fig. 7.

**Fig. 7:**
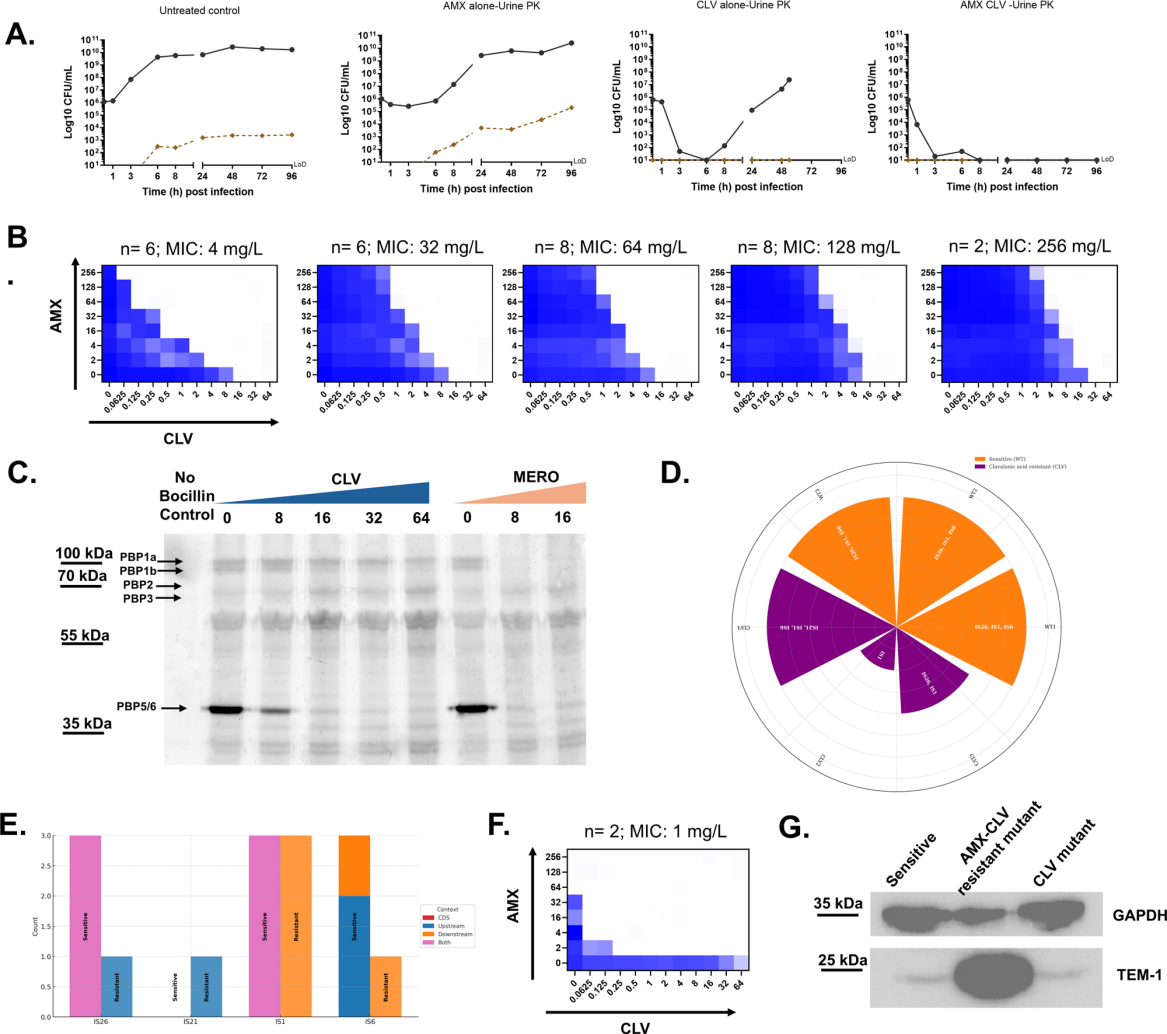
Urinary pharmacokinetic simulations reveal sustained bacterial clearance and distinct resistance mechanisms under high AMX-CLV exposure. **A** Time-kill curves from HFIM simulations mimicking urinary pharmacokinetics of AMX and CLV. Black lines denote total bacterial counts (CFU/mL); orange lines represent resistant subpopulations detected on AMX-CLV-supplemented plates. AMX monotherapy allowed regrowth of resistant subclones, CLV alone produced transient killing followed by regrowth, and the AMX–CLV combination achieved complete sterilisation without resistance emergence. **B** Checkerboard assays showing inhibition profiles of resistant isolates spanning AMX-CLV MICs of 4–256 mg/L. Synergistic inhibition was observed across combinations, while very high CLV concentrations alone achieved bactericidal activity, consistent with the intrinsic antibacterial effects of CLV at urinary exposure levels. **C** Competitive bocillin-binding assay demonstrating concentration-dependent binding of CLV to PBPs 1b, 2, and 5/6 at ≥ 40 mg/L. Meropenem (MERO) served as a positive control for PBP binding. Results are representative of three independent experiments with similar outcomes. **D** Circular mapping of insertion sequence (IS) family distributions flanking *blaTEM-1* in untreated (orange) and CLV-selected (purple) isolates. CLV-selected isolates lacked the expanded IS-mediated transposition unit architectures characteristic of AMX-CLV-selected resistant populations. **E** Relative abundance of IS families (IS26, IS21, IS1, IS6) across genomic contexts in sensitive and resistant isolates, illustrating reduced IS diversification under CLV selection alone. **F** Checkerboard analysis of CLV-selected isolates (MIC 1 mg/L) showing susceptibility to AMX-CLV combination therapy despite partial tolerance to CLV monotherapy. **G** Western blot quantification of TEM-1 protein expression in sensitive, AMX-CLV-selected resistant, and CLV-selected isolates. CLV-selected isolates displayed basal TEM-1 levels comparable to sensitive controls, confirming that resistance under CLV monotherapy is independent of β-lactamase overproduction. Results are representative of three independent experiments with similar outcomes.

AMX monotherapy resulted in rapid bacterial regrowth, and expanding resistant subpopulations, consistent with the selection of *blaTEM-1* overexpressing lineages (Fig. 7A). In contrast, CLV monotherapy produced a rapid ≥3 log_10_ reduction in CFU within 6 h, but regrowth occurred after 24 h despite the absence of colonies on AMX-CLV containing plates. This pattern indicated that regrowth in the CLV arm was not driven by β-lactamase-mediated resistance. Given that the MIC of CLV against strain B50 was 16 mg/L, these data suggested that at high urinary concentrations, CLV may exert intrinsic antibacterial activity through partial inhibition of penicillin-binding proteins (PBPs).

Checkerboard assays using resistant isolates with AMX-CLV MICs ranging from 4 to 256 mg/L confirmed two distinct clearance mechanisms (Fig. 7B, supplementary Fig. 8). At moderate concentrations, bacterial killing depended on the synergistic interaction between the β-lactam and the β-lactamase inhibitor; at very high CLV concentrations, direct antibacterial activity of CLV was observed. Competitive bocillin-binding assays confirmed that CLV binds PBP1b, PBP2, and PBP5/6 at concentrations ≥40 mg/L (Fig. 7C, see supplementary Fig. 9), consistent with its bactericidal activity in the urinary-exposure arm. Meropenem was used as a positive control to confirm assay performance^13,14^.

Whole-genome analysis revealed that isolates selected on CLV alone experiment lacked the IS-mediated *blaTEM-1* amplifications characteristic of AMX-CLV-selected resistant subpopulations (Fig. 7D). These CLV-resistant isolates exhibited minimal structural rearrangement around *blaTEM-1* and no expansion of transposition unit endpoints, suggesting alternative, non-β-lactamase mechanisms of reduced susceptibility potentially involving PBP adaptation (see supplementary Fig. 10). Consistent with this, Western blot analysis showed that CLV-resistant isolates expressed TEM-1 at levels comparable to those of untreated controls, whereas combination-selected resistant clones displayed marked TEM-1 overexpression (Fig. 7E).

In the combination therapy arm of the urinary PK simulation, sustained bacterial eradication was achieved within 24 h with no regrowth or emergence of resistance up to 96 h (Fig. 7A). Together, these findings indicate that high urinary exposures of both AMX and CLV achieve sterilising activity through complementary mechanisms: CLV exhibits intrinsic antibacterial activity at high concentrations consistent with PBP binding, while AMX augments this activity and prevents the survival of any CLV tolerant cells. This pharmacokinetic context uniquely enables AMX-CLV to clear TEM-1-producing *E. coli*, explaining its clinical efficacy in urinary tract infections but limited success at other infection sites.

## Discussion

Our study reveals a coordinated network of molecular and pharmacodynamic processes underpinning the emergence of high-level resistance to AMX-CLV in *E. coli* expressing TEM-1 β-lactamase. When plasma pharmacokinetics were simulated in the HFIM, the rapid selection and expansion of pre-existing resistant subpopulations suggest that the rapid emergence of resistance could contribute to treatment failure. This dynamic contrasts with the sustained bacterial clearance achieved following urinary pharmacokinetic profiles.

The emergence of resistance following plasma concentration-time profiles was driven by the selection of rare, stress-tolerant subpopulations with elevated *blaTEM-1* expression. These cells exhibited oxidative-stress-associated mutations and heightened transposase activity, creating conditions conducive to IS-mediated gene amplification. This adaptive network links oxidative stress, SOS activation, and mobile-element dynamics, leading to rapid amplification of β-lactamase determinants. Such findings provide a mechanistic explanation for the limited efficacy of AMX-CLV for diseases caused by *E. coli* outside the urinary tract.

In this context, our findings can be directly compared with prior studies of β-lactam heteroresistance. Previous work has established heteroresistance to β-lactams as a population-level phenomenon in which a minority subpopulation survives antibiotic exposure and can expand under selection^15^. Notably, Band and Weiss proposed that heteroresistance may often represent an intermediate evolutionary stage preceding stable resistance. Our findings are consistent with this model and extend it by providing a quantitative, cell-level mechanism that can generate such resistant subpopulations. Specifically, we show that heterogeneity in β-lactamase copy number can arise within clonal populations, producing cells with transiently elevated resistance. This dynamic gene amplification provides a plausible molecular basis for heteroresistance observed in population analysis studies and supports the idea that β-lactamase copy number variation may facilitate progression toward stable resistance under continued antibiotic pressure.

β-lactamases remain the most common cause of resistance to penicillins^16,17^. β-lactam/β-lactamase inhibitor combinations, such as AMX-CLV, were developed to extend the therapeutic utility of β-lactams by counteracting β-lactamases^18–20^. Previous studies typically viewed TEM-1-mediated resistance as a static process arising from promoter upregulation or inhibitor-resistant TEM (IRT) substitutions^21–23^. In contrast, our data reveal a dynamic amplification mechanism, mediated by IS-driven translocatable units, that enables rapid shifts in gene copy number and expression without requiring stable mutations.

**Fig. 8:**
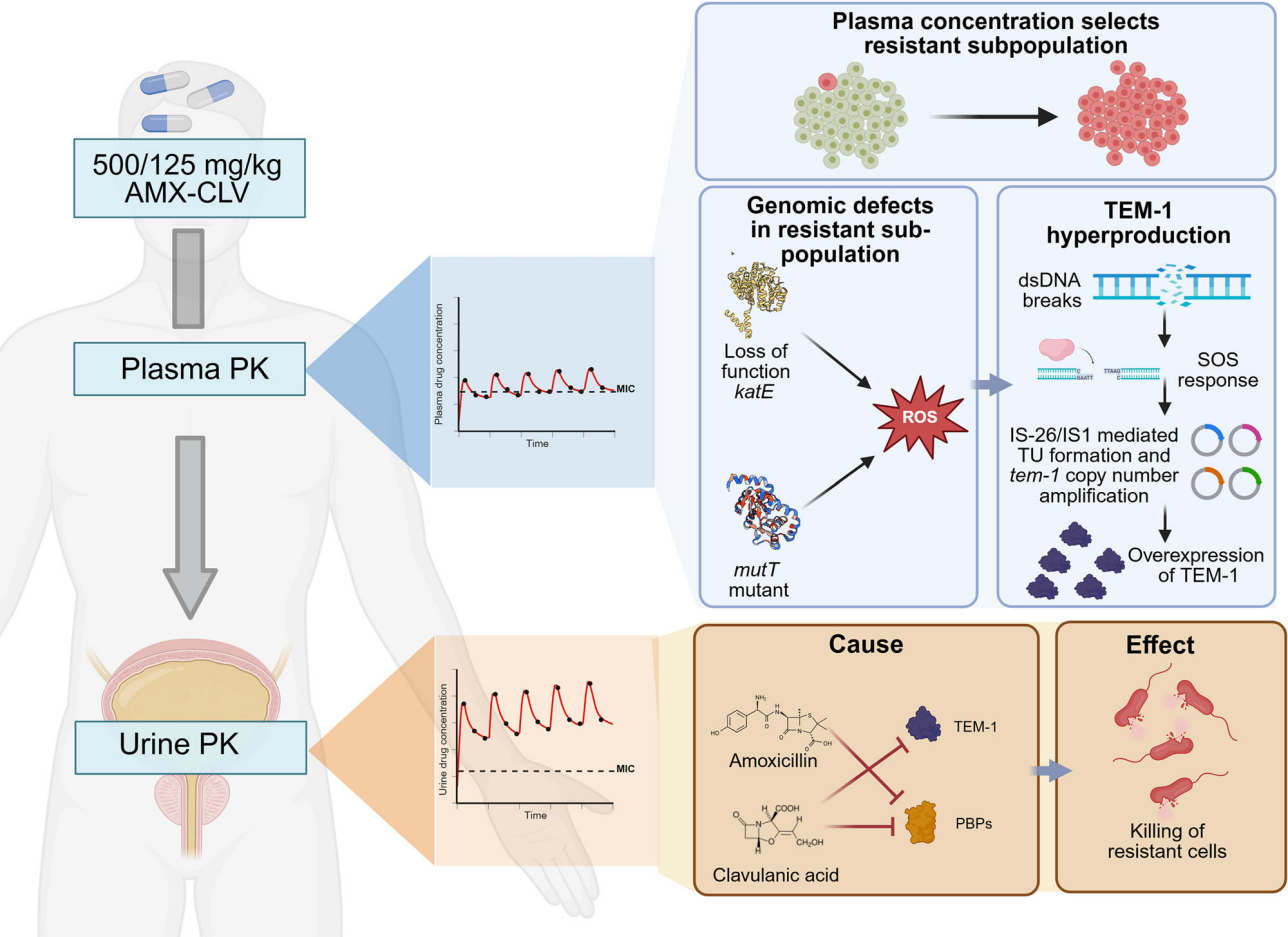
Schematic overview of plasma PK vs urine PK of AMX/CLV. Plasma pharmacokinetics of AMX/CLV select pre-existing resistant *E. coli* subpopulations. Genomic defects (*katE* and *mutT* loss-of-function) drive ROS accumulation and hypermutation, leading to activation of the SOS response and IS-mediated amplification of the *TEM-1* copy number. Elevated β-lactamase production facilitates resistance despite systemic therapy. However, urinary drug concentrations enable AMX/CLV to target susceptible and low-level resistant cells, mediating bacterial clearance. *Created in BioRender. Farrrington, N. (2026)* https://BioRender.com/ohk06z4.

Loss-of-function variants in *mutT* and *katE* may contribute to increased mutagenesis and oxidative imbalance, respectively, and facilitate the accumulation of genetic diversity even before antibiotic challenge. Elevated ROS and SOS activation further mobilises IS elements, driving the observed *blaTEM-1* amplification and structural rearrangements. Together, these events explain the rapid adaptability of TEM-1-producing *E. coli* (Fig. 8).

From a clinical perspective, our findings complement recent EUCAST breakpoint revisions that effectively restrict oral AMX-CLV use against *E. coli* to urinary tract infections^9^. This policy reflects the inability of systemic exposures achievable with standard regimens to meet pharmacodynamic targets required for bacterial killing. Our data provide a mechanistic rationale for this policy and underscore additional complexity introduced by resistance amplification in different tissue subcompartments.

In contrast, urinary pharmacokinetic simulations demonstrated sustained eradication of both sensitive and resistant subpopulations. This outcome was largely attributable to the direct antibacterial activity of CLV, which at high concentrations inhibits PBPs and complements the bactericidal effect of AMX. Although the intrinsic antibacterial activity of CLV has been previously recognised^13^, our data demonstrate PBP1b, PBP2, and PBP5/6 binding at concentrations achievable in urine. The direct antibacterial activity of CLV explains why treatment of urinary infections with AMX-CLV often succeeds.

Isolates selected under CLV monotherapy lacked IS-mediated *blaTEM-1* amplification, supporting the notion that resistance under urinary conditions arises through alternative, non-β-lactamase pathways, possibly involving PBP modification. These findings underscore that high local CLV exposure suppresses the evolutionary routes leading to β-lactamase amplification, stabilising treatment success in the urinary tract.

While the HFIM enables precise dissection of pharmacokinetic and bacterial evolutionary dynamics, it does not incorporate host immune responses. In vivo, immune-mediated clearance is expected to synergise with antibiotic killing, particularly in settings where drug exposures are already sufficient to suppress resistant subpopulations, such as the urinary tract. Under systemic plasma exposures that permit survival and expansion of stress-tolerant cells, immune pressure alone may be insufficient to prevent resistance amplification. Thus, our findings define antibiotic-driven evolutionary trajectories that would operate alongside, rather than independently of, host immunity.

Beyond β-lactamase inhibition, our study highlights broader implications for resistance prevention. Targeting the underlying stress responses and transpositional activity that drive rapid adaptation could represent a new avenue for drug development. Combining β-lactamase inhibitors with compounds that limit SOS induction or inhibit transposition may help forestall the emergence of resistance. Finally, these data underscore the need for diagnostics that assess not only the presence of resistance genes but also their copy number and mobility potential, parameters that more accurately predict resistance trajectories under different pharmacokinetic environments.

## Methods

### Strains and reagents

A total of 30 clinical *E. coli* isolates were obtained from a large tertiary clinical microbiology laboratory serving the city of Liverpool, UK (Liverpool Clinical Laboratories, UK). All isolates were originally stored at −80 °C in cryopreservation beads. For recovery, a single bead was aseptically removed and streaked onto cation-adjusted Mueller-Hinton (CAMH) agar plates, then incubated overnight at 37 °C. The following day, a single well-isolated colony was selected to prepare a glycerol stock (15% glycerol), which was archived at −80 °C.

For all in vitro assays, isolates were revived from the archived glycerol stocks by streaking onto fresh CAMH agar and incubating overnight at 37 °C. A single colony from this plate was then inoculated into broth and grown to the appropriate phase before MIC or other susceptibility testing. All pure and pharmacological grade antibiotics used for MIC testing were purchased from Sigma-Aldrich (Merck, USA). Media and agar used for growth in all experiments were purchased from Merck.

### In vitro susceptibility testing

MIC testing was performed using broth microdilution assay in 96-well round-bottom plates (Corning, USA), according to EUCAST guidelines. Strains were grown in cation-adjusted Mueller-Hinton (CAMHB II) broth (Merck, USA) at a total volume of 200 μL per well. AMX was serially diluted two-fold, with the concentration of CLV fixed at 2 mg/L. The inoculum culture used was maintained at 10^5^ CFU/mL. The plates were incubated at 37 °C for 20–24 h. MIC for all strains was performed in three technical replicates.

### Simulation of human pharmacokinetics

Plasma pharmacokinetic parameters and drug exposure profiles for orally administered AMX-CLV were derived from published data^24^. Replication of this drug exposure profile was simulated using HFIM-like conditions in ADAPT 5 to confirm recapitulation of the pharmacokinetic profile, before simulating in the HFIM^25^.

Urinary pharmacokinetics were predicted using physiology-based pharmacokinetic (PBPK) models built in PK-SIM® (Open Systems Pharmacology) known physical-chemical and pharmacological data for both AMX and CLV, and using available published plasma and urinary time-concentration data for both. With acceptably performing PBPK models, simulations of urinary drug concentrations following oral administrations of AMX/CLV 500/125 mg q8h, with q8h frequency micturition, were simulated. Using ADAPT, drug infusion parameters were identified that approximated the rate of urinary drug accumulation in an HFIM, along with temporary high media pump settings that would approximate urinary drug clearance through micturition.

### Hollow fibre infection model

The HFIM experiments were performed as previously described with minor adjustments^26^. Each arm of the HFIM setup is demonstrated in Supplementary Fig. 11. CAMHB II was continuously pumped into the central compartment at a rate to simulate the physiological clearance of both drugs. In the central compartment of the HFIM assembly, 200 mL of CAMHB II was maintained via elimination pumps. The target simulated half-lives for plasma AMX and CLV were 1.35 h^24^. The urinary pharmacokinetic profile was simulated by infusing the drug over 4 h, with an experimental half-life within the HFIM model of 1 h, to replicate the urinary drug exposure profile simulated by PBPK modelling. Both drugs have negligible protein binding, so no adjustment accounting for this was needed^27^.

All experiments were performed as per previously published standard HFIM experiments^28^. Each arm of the HFIM assay was run for up to 48–96 h, depending on the time we observed treatment failure, i.e., when the resistant sub-population was almost equivalent to the total population. The cartridge (Fibre-Cell Systems Inc.; Cat no: c2011; Polysulfone material; ECS vol: 20 mL) of HFIM was infected with strains at 10^5^ CFU/mL. Each drug was administered into the central compartment every 8 h till the end of the study. Samples were taken for bioanalysis from the central compartment during the first dosing interval, and during steady state later in the experiment to confirm predicted pharmacokinetic profiles were successfully achieved. For quantification of the total bacterial population, samples were collected from the hollow-fibre cartridge at 0, 1, 3, 6, 8, 24, 48, 72, and 96 h (the experiment was terminated when the resistant population reached the total population level). Samples were serially diluted 1:10 in PBS, and 100 μL of each sample and its dilution were plated onto drug-free and drug-containing Mueller-Hinton Agar (MHA) plates. Drug-containing plates contain 32 mg/L AMX and 2.5 mg/L CLV (corresponding to CLV peak plasma concentrations).

### Bioanalysis

Both AMX and CLV (Cambridge Biosciences, UK) were extracted from Mueller-Hinton broth and analysed as follows. The internal standards, [13C6] AMX (Alsachim, France) and sulbactam (Cambridge Biosciences, UK) were prepared in acetonitrile (1 mg/L, Fisher Scientific UK) and 150 uL was added to a 96-well protein precipitation plate [Phenomenex, Cheshire, UK]. Broth sample, blanks, calibrators (*n* = 2 of each level) in the range 0.1–25 mg/L for AMX and 0.25–25 mg/L for CLV and quality controls (*n* = 2 of each level, 0.75, 3.75 and 12.5 mg/L) were mixed with the internal standard on a plate shaker for 5 min at 800 rpm. Liquid was drawn through the protein precipitation plate into a collection plate using a positive-pressure manifold. Samples were then dried down under nitrogen for 45 min, followed by reconstitution with water + 0.1% formic acid (150 uL). The plate was sealed and placed on a plate shaker for 10 min at 800 rpm before being transferred to the autosampler for LC-MS/MS analysis.

LC-MS/MS analysis was carried out using a Waters Acquity UPLC coupled to a Waters Xevo TQXS triple quadrupole mass spectrometer fitted with an electrospray source. The LC-MS system was controlled using Mass Lynx Data Acquisition software (Ver 4.2). Analytes were injected (3 uL) onto a Waters Acquity UPLC HSS T3 (2.1 mm × 100 mm, 1.8 µm) and separated over 4.5 min, gradient using a mixture of solvents A and B. Solvent A was LC-MS grade water with 0.1% (v/v) formic acid. Solvent B was LC-MS grade acetonitrile with 0.1% (v/v) formic acid. Separations were performed by applying a gradient of 5% to 95% solvent B over 3 min, at 0.4 mL/min followed by an equilibration step (1.5 min at 5% solvent B).

The mass spectrometer was operated in negative ion mode using Multiple Reaction Monitoring (MRM). Following an optimisation process, the following mass transitions and collision energies were used for the analysis: 364.2 > 223.1 (Ce 10 eV) for AMX, 370.2 > 229.1 (Ce 12 eV) for 13C6 AMX, 198.1 > 136.1 (Ce 8 eV) for CLV and 232.1 > 140.1 (Ce 12 eV) for sulbactam. The resultant data were processed using Target Lynx processing software (Ver 4.2).

Prior to sample analysis, the analytical method was validated to assess recovery and matrix effects, interday and intraday accuracy and precision, carryover, dilution integrity, stability in the matrix (4 h at room temperature and three freeze-thaw cycles), and processed sample stability (reinjection of extracts after 24 h).

The LLQ was defined as 0.1 mg/L and the LOD 0.05 mg/L for AMX and 0.25 mg/mL and 0.1 mg/L for CLV. For AMX, the inter- and intra-day %CV across the three QC levels ranged from 3.73% to 7.21% and 5.26% to 11.58%, respectively. For CLV, the inter- and intra-day %CV on the three QC levels ranged from 8.55%–13.53% and 8.36%–12.75%, respectively. Both analytes were found to be stable in all conditions described above.

### Population Analysis Profiling (PAP) assay

The bacterial strain was revived from a glycerol stock by streaking onto an MHA plate and incubating at 37 °C overnight. A single colony was then picked and inoculated into 5 mL of Mueller-Hinton broth, incubated at 37 °C with shaking at 200 rpm for 24 h. Subsequently, 1 µL of the 24-h culture was subcultured into 1 mL of fresh Mueller-Hinton broth and incubated under the same conditions for another 24 h. Drug-containing MH agar plates were prepared with concentrations ranging from 0 to 256 µg/mL in twofold dilutions (e.g., 0, 2, 4, 8… 256 µg/mL). Serial dilutions of the overnight culture were prepared and plated onto both drug-free MH plates and drug-containing MH plates, which were incubated at 37 °C for 24–48 h. Colony-forming units (CFU) were counted on each plate, and the survival percentage at each drug concentration was calculated using the formula:

$${Survival}\,{percentage}=\left(\frac{\frac{{CFU}}{{mL}}{in}\,{drug}\,{containing}\,{plate}\left(s\right)}{\frac{{CFU}}{{mL}}{in}\,{drug}\,{free}\,{plate}}\right)*100$$

The resistance profile was analysed by plotting survival percentage against drug concentration.

### Intracellular ROS quantification

The bacterial samples were adjusted to an OD_600_ of 0.2. In microcentrifuge tubes, 1 mL of bacterial samples was exposed to 100 μM DCF-DA (Invitrogen, UK) and incubated at 37 °C with shaking at 200 rpm for 20 min. 100 μL of the sample was added to Blackwell 96-well plates (Corning, USA), and the fluorescence was measured at excitation and emission wavelengths of 485 nm and 528 nm, respectively (Thermo Scientific, Varioskan LUX, USA). The assay was performed with three independent biological replicates, each comprising two technical replicate measurements.

### Real-time qPCR

Total RNA was extracted from bacterial cells using the standard TRIzol (Sigma-Aldrich, Cat no: 93289) protocol. Briefly, 1 μg of total RNA was used to prepare cDNA using the SuperScript III first-strand synthesis kit (NEB, UK). Real-time PCR was performed with the SYBR Green Master Mix (Applied Biosystems) according to the manufacturer’s instructions. Measurements were performed using the QuantStudio 6 × real-time PCR system (Applied Biosystems) with the following conditions: 95 °C for 10 min, 40 cycles of 95 °C for 15 s and 60 °C for 1 min, and a final dissociation cycle of 95 °C for 2 min, 60 °C for 15 s, and 95 °C for 15 s. The primers used are 16S rRNA: = Forward primer: CTCCTACGGGAGGCAGCA; Reverse primer: GWATTACCGCGGCKGCTG TEM-1: = Forward primer: GTCCTCCGATCGTTGTCAGAA; Reverse primer: GCATCTTACGGATGGCATGA. Relative gene expression was calculated using the ΔΔCT method, with 16S rRNA as the reference gene. Experiments were performed in biological duplicates and measured in technical triplicates.

### TUNEL assay

The TUNEL assay was performed using the NEB Live/Dead TUNEL kit (NEB, UK) according to the manufacturer’s instructions. The cells were resuspended in 1% paraformaldehyde in PBS (pH 7.4) at a concentration of 10^6^ cells/ml and incubated on ice for 20 min. The cells were washed with PBS three times, then resuspended in 70% ice-cold ethanol and allowed to stand on ice for 30 min. For staining, the cell pellet was resuspended in 1 ml wash buffer. After two washes, the pellet was incubated in 50 μl of TUNEL reaction mixture containing FITC-dUTP and deoxynucleotidyl transferase at 37 °C in the dark for 1 h. After incubation, cells were washed twice with rinse buffer and incubated with 5 mg/mL BSA. Samples were washed and then resuspended in PBS for fluorescence-activated cell sorting (FACS) analysis. The FITC signal was analysed with an excitation laser at 488 nm and a 525/15 nm bandpass filter using a Cytomaster flow cytometer (BD Biosciences) with a 70-μm nozzle. Graphs were generated using FCS 7 research edition software.

### Cell sorting

Overnight culture of *E. coli* B50 was treated with 0.1 mg/mL of lysozyme at 37 °C for 1 h, followed by three washes with 20% glycerol: PBS solution. Cells were incubated with 2% BSA for 20 min, then incubated with 1:10000 anti-mouse TEM-1 antibody (Santa Cruz Biotechnology, Cat no: sc-66062). After three washes with 20% glycerol in PBS, the cells were incubated with a 1:1000 secondary anti-mouse goat FITC-conjugated antibody (Invitrogen, Cat no: 31547). The cell suspension was passed through the ARES III Cell Sorter instrument. This experiment was repeated twice to gate cells based on low and high fluorescence. The sorted cells were serially diluted, plated onto MH agar, and incubated overnight at 37 °C. Ten colonies from each group were selected and tested for MIC determination.

### DNA read processing and variant detection

Raw Illumina paired-end reads were processed by the sequencing facility (Centre for Genomic Research, University of Liverpool) using a standardised whole-genome variant detection workflow. Adaptor-trimmed and quality-filtered read pairs were aligned to the appropriate reference genome (*E. coli* MG1655) using BWA-MEM v0.7.17^29^. SAM/BAM alignment files were subsequently filtered with SAMtools v1.10 to remove unmapped reads, secondary and supplementary alignments, and reads with a mapping quality <10^30^.

PCR duplicate reads were identified and marked using Picard Tools v2.23.3 (Broad Institute). Following duplicate removal, high-confidence read alignments were subjected to variant calling using the Genome Analysis Toolkit (GATK) v4.2^31,32^. Single-nucleotide polymorphisms (SNPs) and small insertions/deletions (indels) were identified using the HaplotypeCaller module following GATK Best Practices.

Variants were subjected to hard filtering based on GATK recommendations to minimise false positives. For SNPs, filtering thresholds included: QD < 2.0, FS > 60.0, MQ < 40.0, SOR > 3.0, MQRankSum <–12.5, and ReadPosRankSum <–8.0. For indels, QD < 2.0, FS > 200.0, QUAL < 30.0, and ReadPosRankSum <–20.0 were applied. High-confidence variants passing all filters were annotated using SnpEff v4.2^33^, providing predicted functional effects on protein-coding genes.

### Genome assembly and functional annotation

To generate a draft genome assembly for downstream analyses, quality-filtered reads were assembled de novo using SPAdes v3.15.4 with the *--isolate* mode and default parameters^34^. Contigs <500 bp or with <5 × coverage were removed to avoid spurious assembly.

Gene prediction and functional annotation of assembled contigs were performed using Bakta v1.8, which provides standardised annotation of protein-coding genes, RNAs, operons, and prophage-associated features^35^.

Detection of antimicrobial resistance (AMR) determinants and resistance-associated variants was performed using the Resistance Gene Identifier (RGI) v6.0.0 with default parameters and the CARD database (version current at analysis)^36^. Perfect and strict hits were retained for the interpretation of resistance gene content.

### Mobile genetic element and insertion sequence detection

Insertion sequences (IS elements) and mobile genetic elements were identified using three complementary tools. ISEScan v1.7.2.3 was used for genome-wide prediction of IS families based on HMM profiles^37^. ISfinder was used to assign IS family identity based on nucleotide similarity^38^. MobileElementFinder v1.0.3 was run on assembled contigs using default parameters to annotate mobile elements, insertion sites, and associated genetic cargo^39^. This combined approach maximises detection sensitivity and classification accuracy.

### RNA-seq read processing and differential gene expression analysis

RNA sequencing libraries were processed by the facility using a reference-based transcriptomic workflow. Quality-checked reads were aligned against the corresponding reference genome (*E. coli* strain JE86-ST05**;** NCBI Reference Sequence: NZ_AP022815.1) using the splice-aware aligner HISAT2 v2.2.1^40^. Alignment was performed using default parameters optimised for bacterial gene structures (no introns assumed).

Aligned reads were quantified using HTSeq v2.0 in stranded mode, based on gene models defined in the corresponding GFF3 annotation file^41^. Genes with low counts were filtered using the filterByExpr function in edgeR to remove features lacking sufficient read coverage for statistical testing^42^.

Differential expression analysis was performed using DESeq2 v1.34.0^43^. Gene-wise dispersion estimates were fit using empirical Bayes shrinkage, and pairwise contrasts were evaluated using Wald tests with Benjamini-Hochberg correction for multiple comparisons. Genes with adjusted *P* < 0.05 and |log_2_ fold change| > threshold (insert threshold used in your study) were considered differentially expressed.

### PacBio HiFi Long-read processing and plasmid assembly

HiFi long-read sequencing data were processed by the Centre for Genomic Research (University of Liverpool). Circular plasmid-enriched DNA was sequenced using PacBio HiFi chemistry, and high-accuracy reads were assembled de novo using Hifiasm (v0.19.5) in HiFi mode with default parameters^44^. For each sample, polished contigs representing plasmid candidates were retained for downstream analysis. Samples that did not generate any Hifiasm contigs (19.3_3 and 25.8_8) were excluded.

### Clustering of parental plasmid contigs

To establish a non-redundant parental plasmid reference, Hifiasm-assembled contigs from the three untreated parental samples (Set 1–3) were clustered using CD-HIT (v4.8.1) with default nucleotide settings^45^. This produced a representative set of “parental plasmid contigs” used as the baseline for all comparative analyses.

### Identification of Closest Reference Plasmids (PLSDB Search)

Representative parental contigs were compared against the PLSDB plasmid database using MASH (v2.3) with sketching parameters *p* = *0.1* and *d* = *0.1*^46^. For each contig, the closest high-identity PLSDB plasmid was identified, and its corresponding FASTA sequence was downloaded. These sequences served as “PLSDB parent references” for structural variant (SV) detection against known plasmid backbones.

### Long-read structural variant detection

SV detection was performed using complementary long-read callers, pbsv (PacBio), following standard long-read SV calling workflows^47^. Three levels of comparison were performed: Parent vs PLSDB reference; Parent plasmid contigs were aligned to their closest PLSDB plasmid using minimap2 (v2.26), and SVs were identified using pbsv to define baseline plasmid structure^48^. Derived (resistant) vs PLSDB reference; Derived-sample plasmid contigs were aligned against the same PLSDB references to detect SVs associated with antibiotic-selected evolution. Derived vs clustered parental contigs (direct comparison); To identify de novo structural changes emerging under selection, contigs from derived samples were aligned directly against the clustered parental contigs. SV calling in this comparison captured insertions, deletions, rearrangements, duplications, and cassette movements relative to the ancestral plasmid background. The output for each comparison included: BAM alignments, FASTA/FAI references, and VCF files of SVs (importable into IGV). VCF files with no records indicated that no structural variants were detected.

### Identification of rare and horizontally acquired plasmids

To identify low-abundance plasmid elements potentially acquired during evolution, contigs were screened using a distance vs coverage approach. MASH distance was plotted against read depth; contigs with *distance* < *0.1* but *coverage* ≤ *30 ×* were classified as high-identity, low-copy plasmids. Unique, rare plasmid contigs were summarised (94 total).

### Analysis of *blaTEM-1* genetic context diversity

The genomic environments surrounding *blaTEM-1* were analysed to determine whether the observed diversity of insertion sequence (IS) arrangements deviated from random expectation. For each contig containing *blaTEM-1*, flanking IS elements were identified using outputs from ISEScan, ISFinder, and MobileElementFinder, and their genomic coordinates were parsed to reconstruct ordered gene contexts (e.g., *IS26-blaTEM-1-IS1*), retaining relative orientation and adjacency.

To quantify empirical context diversity, a bootstrap-based sampling approach was applied. Let *N* denote the total number of *blaTEM-1-*containing contigs. For each sampling depth *n ∈ [1, N]*, *n* contexts were sampled with replacement, and the number of unique structural contexts was recorded. This procedure was repeated 100 times per *n* to generate bootstrap estimates of diversity accumulation curves.

A null model was generated to evaluate whether observed diversity exceeded expectations under independent IS insertions. Valid IS-*blaTEM-1* arrangements were simulated combinatorially, allowing each IS family to appear at most once on either side of *blaTEM-1*, with symmetric wrapping permitted only where biologically plausible. As with the empirical model, *n* simulated contexts were sampled with replacement across 100 bootstrap replicates to obtain a null distribution of expected diversity.

Median and interquartile ranges from both empirical and null models were compared to assess whether the structural diversity observed in the dataset was greater than expected under independent IS insertion dynamics. All scripts used to perform these simulations are available at: https://github.com/agerada/tem-1-bootstrap.

### Checkerboard assays

Checkerboard assays were performed to assess the interaction between AMX and CLV over a two-dimensional concentration range. AMX and CLV stock solutions were freshly prepared in sterile DMSO and water (filter-sterilised (0.22 µm)(Millex-CV)). Two-fold serial dilutions of each compound were prepared in CAMHB II to generate working solutions spanning the concentration range for each isolate in 100 µL. For plate preparation, AMX dilutions were dispensed horizontally across 96-well microtiter plates, while CLV dilutions were dispensed vertically, creating a two-dimensional matrix of drug combinations. Growth-control wells (no drug) and sterility controls (no inoculum) were included on every plate. Bacterial isolates were grown overnight at 37 °C with shaking at 200 rpm in 10 mL CAMHB II. Saturated cultures were diluted 1:10,000 into fresh CAMHB II and incubated to an OD₆₀₀ of ~0.4 (mid-log phase). Cultures were subsequently diluted to 10^5^ CFU/mL in CAMHB II, and 100 µL of this inoculum was added to each well of the prepared plates. Plates were incubated statically at 37 °C for 16 h.

### Competitive bocillin binding assay

Cultures were grown in lysogeny broth (LB) at 37 °C with shaking until an optical density at 600 nm of approximately 1.0 was reached, rapidly chilled on ice, and pelleted by centrifugation (5000 × *g*, 10 min, 4 °C) (Eppendorf 5425 R). Cell pellets were resuspended in phosphate-buffered saline (PBS, pH 7.5) and treated sequentially with lysozyme (400 µg mL^−1^, 1 h, 37 °C) and a nuclease mixture containing DNase I and RNase A (10 µg mL^−1^ each) together with an EDTA-free protease inhibitor cocktail. Cells were disrupted using a French pressure cell (Constant System Ltd), and unbroken debris was removed by centrifugation at 12,000 × *g* for 10 min at 4 °C. Membranes were collected from the supernatant by ultracentrifugation (Thermo Scientific Sorval MTX 150) at 150,000 × *g* for 40 min at 4 °C, resuspended in PBS (300–500 µL), aliquoted, and stored at −80 °C. Protein concentration was determined using a BCA assay (Pierce BCA Protein assay kit, Thermo Scientific) with bovine serum albumin standards. For Bocillin FL binding, 17 µg of membrane protein was incubated in PBS (20 µL final volume) with test compounds for 10 min at 30 °C, followed by the addition of Bocillin FL (Thermo Fisher; Cat no.:B13233) (12.5 µM final) and further incubation for 20 min at 30 °C. Reactions were quenched with Laemmli buffer containing freshly added DTT and heated at 95 °C for 3 min before SDS-PAGE. Fluorescently labelled PBPs were visualised using a ChemiDoc gel imager (Bio-Rad) with ProQ-Emerald 488 settings, and total protein was optionally verified by Coomassie staining. Reactions lacking Bocillin FL served as fluorescence controls, those lacking competitor antibiotics defined maximal labelling, and meropenem was used as a positive control to validate expected PBP-binding profiles. Band-intensity comparisons across conditions quantified the competitive inhibition of Bocillin FL binding by AMX and CLV.

### Western blotting

Bacterial cultures were grown in lysogeny broth (LB) to mid-log phase (O.D.₆₀₀ ≈ 1.0) and harvested by centrifugation (5000 × *g*, 10 min, 4 °C). Cell pellets were resuspended in PBS (pH 7.4) containing an EDTA-free protease inhibitor cocktail (Roche Diagnostics, Germany) and lysed by sonication on ice. Lysates were clarified by centrifugation (12,000 × *g*, 10 min, 4 °C), and total protein concentrations were determined using a BCA assay with bovine serum albumin standards. Equal amounts of protein (typically 50 µg per lane) were mixed with Laemmli sample buffer containing DTT, heated at 95 °C for 5 min, and resolved by SDS–PAGE on 12% polyacrylamide gels. Proteins were transferred to nitrocellulose membranes (Amersham; Cat no.: 10600002) (0.45 µm pore size) using a wet-transfer apparatus (100 V, 1 h, 4 °C). Membranes were blocked for 1 h at room temperature in 5% (w/v) skimmed milk in Tris-buffered saline containing 0.1% Tween-20 (TBST) and incubated overnight at 4 °C with mouse polyclonal anti-TEM-1 antibody (1:5000 dilution in TBST + 1% milk). After three washes in TBST, membranes were incubated with horseradish-peroxidase-conjugated goat anti-mouse IgG secondary antibody (Abcam; Cat no.: ab6728) (1:10,000, 1 h, room temperature). Loading control GAPDH (Invitrogen; Cat no.: MA5-15738) was used. Bands were visualised using enhanced chemiluminescence (ECL) substrate (Amersham) and imaged with a digital chemiluminescence detection system.

### Statistics and reproducibility

Sample sizes for all experiments were determined based on established standards in microbiological, pharmacodynamic, and transcriptomic studies, and are consistent with prior HFIM and RNA-seq literature. No formal statistical power calculations were performed. The sample sizes used are sufficient to detect biologically meaningful differences in bacterial growth dynamics, gene expression, and resistance phenotypes.

For all in vitro experiments, the unit of study was an independent bacterial culture. Experiments were performed using multiple independent biological replicates, as specified in the figure legends, with technical replicates included where appropriate.

Samples were not randomly allocated to experimental groups, as all experiments were conducted in controlled in vitro systems using identical starting inocula. Experimental groups were defined solely by treatment conditions (e.g., antibiotic exposure profiles and concentrations), and all variables, including growth conditions, media composition, and incubation parameters, were standardised across groups to minimise potential confounding.

### Reporting summary

Further information on research design is available in the Nature Portfolio Reporting Summary linked to this article.

## Supplementary information

Supplementary Information

Reporting Summary

Transparent Peer Review file

## Source data

Source data

## Data Availability

Sequencing data generated in this study have been deposited in the European Nucleotide Archive (ENA) under the following accession codes PRJNA1378964:[https://www.ncbi.nlm.nih.gov/bioproject/PRJNA1378964]; PRJEB107892:[https://www.ebi.ac.uk/ena/browser/view/PRJEB107892]. All other data supporting the findings of this study are provided in the manuscript, the Source Data file, and the Supplementary Information, including raw numerical values and uncropped, unprocessed scans of blots. The custom code used to analyse blaTEM-1 genetic context diversity is publicly available at GitHub (https://github.com/agerada/tem-1-bootstrap). Source data are provided with this paper.

## Code availability

The custom code used in this study is available at: https://github.com/agerada/tem-1-bootstrap (10.5281/zenodo.19712613).

## References

[1] Kaper, J. B., Nataro, J. P. & Mobley, H. L. T. Pathogenic Escherichia coli. *Nat. Rev. Microbiol.* **2**, 123–140 (2004). 15040260 10.1038/nrmicro818

[2] Ludden, C. et al. Defining nosocomial transmission of Escherichia coli and antimicrobial resistance genes: a genomic surveillance study. *Lancet Microbe* **2**, e472–e480 (2021). 34485958 10.1016/S2666-5247(21)00117-8 PMC8410606

[3] Cantu, C., Huang, W. & Palzkill, T. Cephalosporin substrate specificity determinants of TEM-1 β-lactamase. *J. Biol. Chem.* **272**, 29144–29150 (1997). 9360991 10.1074/jbc.272.46.29144

[4] Lawrence, J. et al. Innovative approaches in phenotypic beta-lactamase detection for personalised infection management. *Nat. Commun.* **15**, 9070 (2024). 39433753 10.1038/s41467-024-53192-7 PMC11494114

[5] Kalp, M. et al. Efficient inhibition of class A and class D β-lactamases by michaelis complexes. *J. Biol. Chem.* **282**, 21588–21591 (2007). 17561511 10.1074/jbc.C700080200

[6] Hubbard, A. T. M. et al. Piperacillin/tazobactam resistance in a clinical isolate of Escherichia coli due to IS26-mediated amplification of blaTEM-1B. *Nat. Commun.* **11**, 4915 (2020). 33004811 10.1038/s41467-020-18668-2 PMC7530762

[7] Brogden, R. N. et al. Amoxycillin/clavulanic acid. *Drugs* **22**, 337–362 (1981). 7037354 10.2165/00003495-198122050-00001

[8] Amoxicillin-clavulanate and Enterobacteriaceae from urinary tract infections. https://www.eucast.org/eucast_news/news_singleview?cHash=6cc831d98b3149393f261d241e60325e&tx_ttnews%5Btt_news%5D=36. (2012).

[9] The European Committee on Antimicrobial Susceptibility Testing (EUCAST). Breakpoint tables for interpretation of MICs and zone diameters, version 15.0. https://www.eucast.org/clinical_breakpoints. (2025).

[10] Qi, W., Jonker, M. J., de Leeuw, W., Brul, S. & ter Kuile, B. H. Reactive oxygen species accelerate de novo acquisition of antibiotic resistance in E. coli. *iScience* **26**, 108373 (2023). 38025768 10.1016/j.isci.2023.108373 PMC10679899

[11] Adam, D., de Visser, I. & Koeppe, P. Pharmacokinetics of amoxicillin and clavulanic acid administered alone and in combination. *Antimicrob. Agents Chemother.* **22**, 353–357 (1982). 7137979 10.1128/aac.22.3.353 PMC183747

[12] Witkowski, G., Lode, H., Höffken, G. & Koeppe, P. Pharmacokinetic studies of amoxicillin, potassium clavulanate and their combination. *Eur. J. Clin. Microbiol.* **1**, 233–237 (1982). 7173186 10.1007/BF02019714

[13] Spratt, B. G., Jobanputra, V. & Zimmermann, W. Binding of Thienamycin and Clavulanic Acid to the Penicillin-Binding Proteins of *Escherichia coli* K-12. *Antimicrob. Agents Chemother.* **12**, 406–409 (1977). 334066 10.1128/aac.12.3.406 PMC429926

[14] Montaner, M. et al. Unravelling the triad of penicillin-binding proteins, β-lactamase activity, and mRNA dynamics. In *Pseudomonas aeruginosa,* AmpC induction. *Journal of Antimicrobial Chemotherapy.* **81**, (2026). 10.1093/jac/dkaf408 PMC12802951 41206063

[15] Band, V. I. & Weiss, D. S. Heteroresistance to beta-lactam antibiotics may often be a stage in the progression to antibiotic resistance. *PLoS Biol.* **19**, e3001346 (2021). 34283833 10.1371/journal.pbio.3001346 PMC8351966

[16] Bradford, P. A. Extended-spectrum beta-lactamases in the 21st century: characterization, epidemiology, and detection of this important resistance threat. *Clin. Microbiol. Rev.* **14**, 933–951 (2001). 11585791 10.1128/CMR.14.4.933-951.2001 PMC89009

[17] Majiduddin, F. K., Materon, I. C. & Palzkill, T. G. Molecular analysis of beta-lactamase structure and function. *Int. J. Med. Microbiol.* **292**, 127–137 (2002). 12195735 10.1078/1438-4221-00198

[18] Bush, K. Past and present perspectives on β-lactamases. *Antimicrob. Agents Chemother.* **62**, e01076–18 (2018). 30061284 10.1128/AAC.01076-18 PMC6153792

[19] Tehrani, K. H. M. E. & Martin, N. I. β-lactam/β-lactamase inhibitor combinations: an update. *Medchemcomm* **9**, 1439–1456 (2018). 30288219 10.1039/c8md00342d PMC6151480

[20] Bush, K. & Bradford, P. A. β-lactams and β-lactamase inhibitors: an overview. *Cold Spring Harb. Perspect. Med.* **6**, a025247 (2016). 27329032 10.1101/cshperspect.a025247 PMC4968164

[21] Zhou, K., Tao, Y., Han, L., Ni, Y. & Sun, J. Piperacillin-Tazobactam (TZP) resistance in Escherichia coli due to hyperproduction of TEM-1 β-lactamase mediated by the promoter Pa/Pb. *Front. Microbiol.* **10**, 833 (2019). 31040841 10.3389/fmicb.2019.00833 PMC6476967

[22] Chaibi, E. B., Sirot, D., Paul, G., Labia, R. & Inhibitor-resistant, T. E. M. lactamases: phenotypic, genetic and biochemical characteristics. *J. Antimicrob. Chemother.* **43**, 447–458 (1999). 10350372 10.1093/jac/43.4.447

[23] Knox, J. R. Extended-spectrum and inhibitor-resistant TEM-type beta-lactamases: mutations, specificity, and three-dimensional structure. *Antimicrob. Agents Chemother.* **39**, 2593–2601 (1995). 8592985 10.1128/aac.39.12.2593 PMC162995

[24] Burkhardt, O. Single- and multiple-dose pharmacokinetics of linezolid and co-amoxiclav in healthy human volunteers. *J. Antimicrob. Chemother.* **50**, 707–712 (2002). 12407127 10.1093/jac/dkf163

[25] David, Z. & D’Argenio, A. S. X. W. ADAPT 5 user’s guide: pharmacokinetic/pharmacodynamic systems analysis software. *Los Angeles: Biomed. Simul. Resour.* **1**, 316 (2009).

[26] Darlow, C. A. et al. Assessment of flomoxef combined with amikacin in a hollow-fibre infection model for the treatment of neonatal sepsis in low- and middle-income healthcare settings. *J. Antimicrob. Chemother.* **77**, 3349–3357 (2022). 36177766 10.1093/jac/dkac323 PMC9704437

[27] Woodnutt, G., Berry, V. & Mizen, L. Effect of protein binding on penetration of beta-lactams into rabbit peripheral lymph. *Antimicrob. Agents Chemother.* **39**, 2678–2683 (1995). 8593001 10.1128/aac.39.12.2678 PMC163011

[28] Sadouki, Z. et al. Application of the hollow fibre infection model (HFIM) in antimicrobial development: a systematic review and recommendations of reporting. *J. Antimicrob. Chemother.* **76**, 2252–2259 (2021). 34179966 10.1093/jac/dkab160 PMC8361333

[29] Li, H. Aligning sequence reads, clone sequences and assembly contigs with BWA-MEM. *arXiv*10.48550/arXiv.1303.3997 (2013).

[30] Li, H. et al. The sequence alignment/map format and SAMtools. *Bioinformatics* **25**, 2078–2079 (2009). 19505943 10.1093/bioinformatics/btp352 PMC2723002

[31] McKenna, A. et al. The genome analysis toolkit: a MapReduce framework for analyzing next-generation DNA sequencing data. *Genome Res* **20**, 1297–1303 (2010). 20644199 10.1101/gr.107524.110 PMC2928508

[32] DePristo, M. A. et al. A framework for variation discovery and genotyping using next-generation DNA sequencing data. *Nat. Genet.* **43**, 491–498 (2011). 21478889 10.1038/ng.806 PMC3083463

[33] Cingolani, P. et al. A program for annotating and predicting the effects of single-nucleotide polymorphisms. *SnpEff. Fly.* **6**, 80–92 (2012). 22728672 10.4161/fly.19695 PMC3679285

[34] Bankevich, A. et al. SPAdes: a new genome assembly algorithm and its applications to single-cell sequencing. *J. Comput. Biol.* **19**, 455–477 (2012). 22506599 10.1089/cmb.2012.0021 PMC3342519

[35] Schwengers, O. et al. Bakta: rapid and standardized annotation of bacterial genomes via alignment-free sequence identification. *Microb. Genom.* **7**, 685 (2021). 10.1099/mgen.0.000685 PMC8743544 34739369

[36] Alcock, B. P. et al. CARD 2023: expanded curation, support for machine learning, and resistome prediction at the Comprehensive Antibiotic Resistance Database. *Nucleic Acids Res.* **51**, D690–D699 (2023). 36263822 10.1093/nar/gkac920 PMC9825576

[37] Xie, Z. & Tang, H. ISEScan: automated identification of insertion sequence elements in prokaryotic genomes. *Bioinformatics* **33**, 3340–3347 (2017). 29077810 10.1093/bioinformatics/btx433

[38] Siguier, P., Filée, J. & Chandler, M. Insertion sequences in prokaryotic genomes. *Curr. Opin. Microbiol.* **9**, 526–531 (2006). 16935554 10.1016/j.mib.2006.08.005

[39] Johansson, M. H. K. et al. Detection of mobile genetic elements associated with antibiotic resistance in *Salmonella enterica* using a newly developed web tool: MobileElementFinder. *J. Antimicrob. Chemother.* **76**, 101–109 (2021). 33009809 10.1093/jac/dkaa390 PMC7729385

[40] Kim, D., Paggi, J. M., Park, C., Bennett, C. & Salzberg, S. L. Graph-based genome alignment and genotyping with HISAT2 and HISAT-genotype. *Nat. Biotechnol.* **37**, 907–915 (2019). 31375807 10.1038/s41587-019-0201-4 PMC7605509

[41] Anders, S., Pyl, P. T. & Huber, W. HTSeq—a Python framework to work with high-throughput sequencing data. *Bioinformatics* **31**, 166–169 (2015). 25260700 10.1093/bioinformatics/btu638 PMC4287950

[42] Robinson, M. D., McCarthy, D. J. & Smyth, G. K. <tt>edgeR</tt>: a Bioconductor package for differential expression analysis of digital gene expression data. *Bioinformatics* **26**, 139–140 (2010). 19910308 10.1093/bioinformatics/btp616 PMC2796818

[43] Love, M. I., Huber, W. & Anders, S. Moderated estimation of fold change and dispersion for RNA-seq data with DESeq2. *Genome Biol.* **15**, 550 (2014). 25516281 10.1186/s13059-014-0550-8 PMC4302049

[44] Wen, H. et al. TRFill: synergistic use of HiFi and Hi-C sequencing enables accurate assembly of tandem repeats for population-level analysis. *Genome Biol.* **26**, 227 (2025). 40721805 10.1186/s13059-025-03685-5 PMC12305924

[45] Fu, L., Niu, B., Zhu, Z., Wu, S. & Li, W. CD-HIT: accelerated for clustering the next-generation sequencing data. *Bioinformatics* **28**, 3150–3152 (2012). 23060610 10.1093/bioinformatics/bts565 PMC3516142

[46] Ondov, B. D. et al. Mash: fast genome and metagenome distance estimation using MinHash. *Genome Biol.* **17**, 132 (2016). 27323842 10.1186/s13059-016-0997-x PMC4915045

[47] Xia, Z. et al. CSV-Filter: a deep learning-based comprehensive structural variant filtering method for both short and long reads. *Bioinformatics* **40**, btae539 (2024). 39240375 10.1093/bioinformatics/btae539 PMC11419953

[48] Li, H. Minimap2: pairwise alignment for nucleotide sequences. *Bioinformatics* **34**, 3094–3100 (2018). 29750242 10.1093/bioinformatics/bty191 PMC6137996

