# Supplementary material for: Molecular pharmacodynamics of amoxicillin-clavulanic acid for urinary tract infections caused by Escherichia coli
Source: Nat Commun. 2026 Jun 13;17:7504. doi: 10.1038/s41467-026-74323-2 (PMC13408132; doi:10.1038/s41467-026-74323-2)
Supplement: Supplementary file 1 — Supplementary Information [file 41467_2026_74323_MOESM1_ESM.pdf]

Supplementary information

Molecular pharmacodynamics of amoxicillin-clavulanic acid for urinary tract infections caused by *Escherichia coli*

Supplementary Table S1. MIC profiles of clinical *E. coli* isolates to AMX alone and in combination with CLV. Minimum inhibitory concentrations (MICs; mg/L) for 27 clinical *E. coli* isolates obtained from blood or tissue samples. MICs for AMX and AMX-CLV were determined by broth microdilution according to EUCAST guidelines. All isolates exhibited AMX MICs >64 mg/L. Addition of CLV resulted in a 2- to 16-fold reduction in MICs. The β-lactamases present in each isolate are listed, including TEM, SHV, CTX-M, OXA, and CMY enzyme families. (EC-5: Enterobacterales Cephalosporinase, family 5)

**Strain (*E. coli*)**	**Source**	**MIC (mg/L)**		**Β-Lactamase(s)**
		**AMX**	**AMX+CLV**	
L10	Blood	>64	32	TEM-1, EC-5
L25	Blood	>64	16	TEM-1, EC-5
L35	Blood	>64	8	SHV-1, EC-5
L50	Blood	>64	8	TEM-1, EC-5
L57	Blood	>64	8	TEM-1, EC-5
L73	Blood	>64	16	TEM-1, EC-5
J13	Blood	>64	16	CTXM-15; OXA-1; CMY-181
J76	Blood	>64	8	TEM-1, EC-5
E21	Blood	>64	16	TEM-1; EC-5
H71	Blood	>64	32	TEM-1
I26	Blood	>64	16	TEM-1, EC-5
I27	Blood	>64	8	TEM-1, EC-5
I35	Blood	>64	32	TEM-1; EC-5
M25	Blood	>64	8	SHV-1, EC-5
M37	Blood	>64	8	TEM-1, EC-5
K10	Blood	>64	32	TEM-1, CMY-181
K11	Blood	>64	8	TEM-1, EC-5
K23	Blood	>64	8	TEM-1
N1	Blood	>64	8	TEM-1, EC-8
N2	Blood	>64	16	TEM-1, EC-5
N4	Blood	>64	8	TEM-1, EC-5
N8	Blood	>64	8	TEM-1, EC-5
N15	Blood	>64	16	TEM-1, EC-14
N16	Blood	>64	16	TEM-1, EC-5
N17	Blood	>64	16	TEM-1, EC-5
N18	Tissue	>64	16	TEM-1, EC-8
N22	Blood	>64	8	TEM-1, EC-5

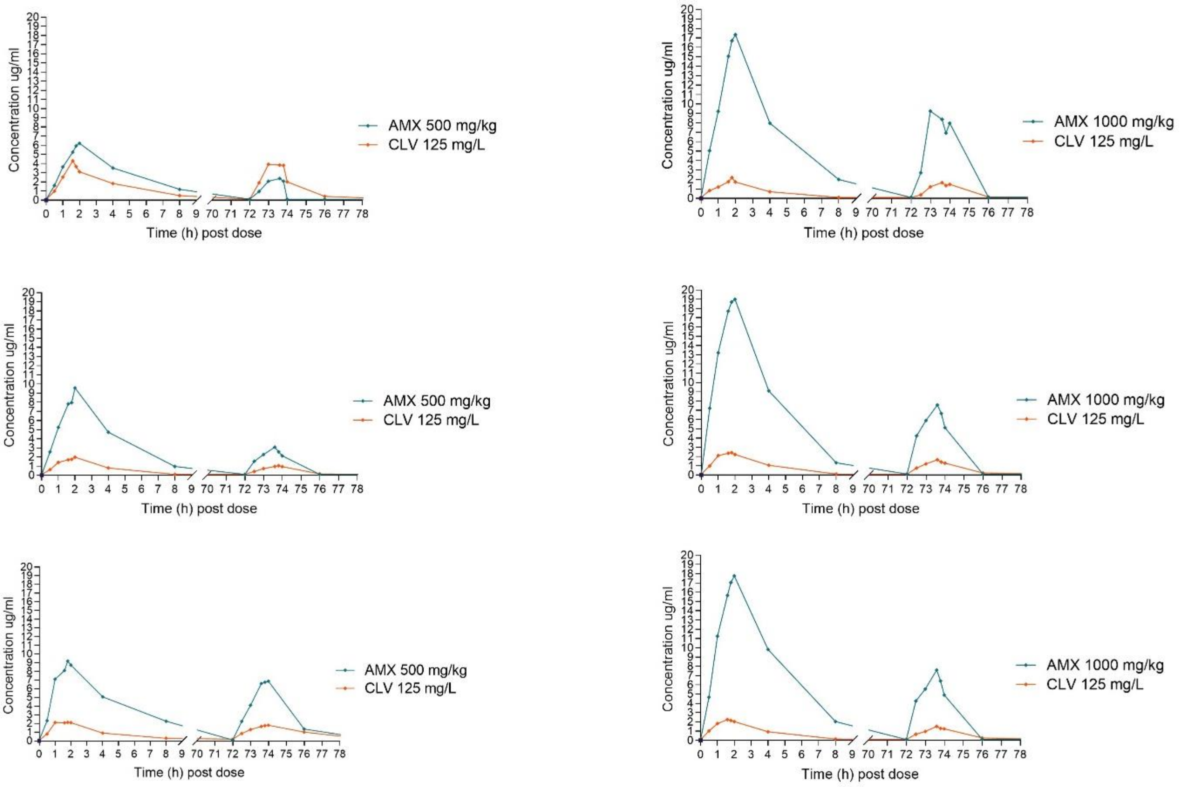

Supplementary Fig. S1. Human-simulated plasma pharmacokinetic profiles of AMX and CLV in the HFIM. Concentration-time profiles for AMX and CLV over 48 h in the hollow-fibre infection model under two dosing regimens: AMX–CLV 500/125 mg every 8 h (left panels) and AMX–CLV 1000/125 mg every 8 h (right panels). Each panel represents one experimental arm, showing AMX (blue) and CLV (orange) concentrations following repeated dosing to reproduce human plasma pharmacokinetics. Drugs were administered every 8 hours throughout the duration of the experiment. Pharmacokinetic sampling was performed on Day 1 and on the day prior to experiment completion. Data represent a single experiment (n = 1 independent HFIM run); concentrations were measured using validated LC-MS/MS methods.

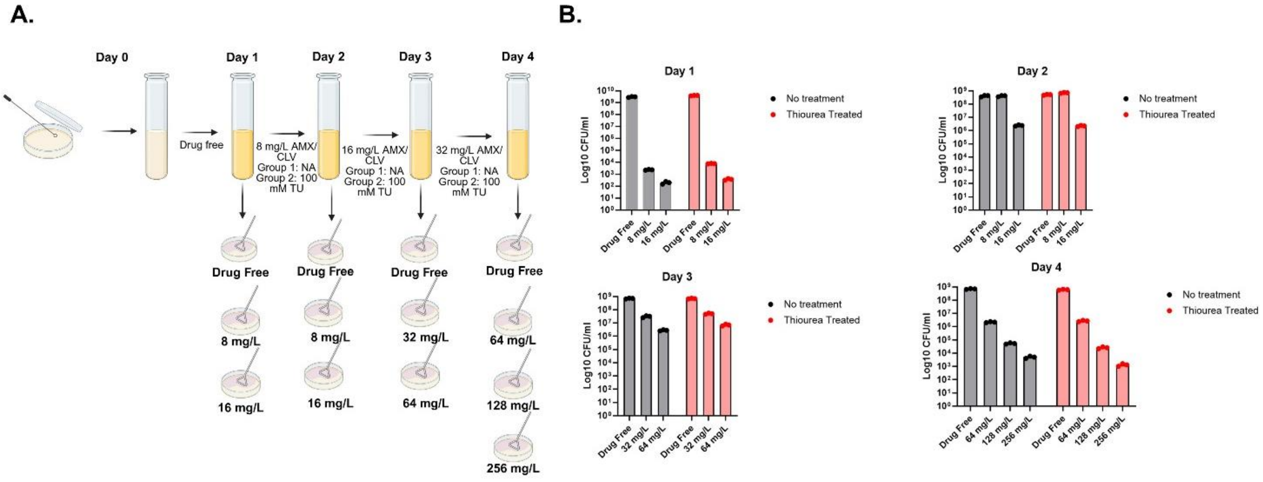

Supplementary Figure S2. Serial passage of *E. coli* B50 under escalating AMX-CLV exposure and assessment of resistance emergence. A. Schematic of the serial-passage experiment in HFIM showing daily sampling, drug-free controls, and stepwise increases in AMX–CLV concentrations from 8/32 mg/L to 64/128 mg/L over four days. Cultures were diluted 1:100 every 24 h and plated on non-selective or AMX-CLV-supplemented agar to quantify total and resistant populations. *Created in BioRender. Farrrington, N. (2026) https://BioRender.com/vwbch2w* **B.** Bacterial densities ($\log_{10}$ CFU/mL) for untreated controls (black) and AMX-CLV-treated populations (red) across Days 1-4. Treated populations showed progressive enrichment of colonies capable of growth on AMX-CLV plates, indicating selection of a drug-tolerant/resistant fraction despite minimal change in total population densities. Data points represent biological replicates (n=3); each bar reflects mean CFU counts on drug-free versus AMX-CLV plates at the indicated time points. These data are presented for descriptive purposes to illustrate trends in resistance emergence; no formal statistical comparisons were performed, and interpretation is based on observed trends across independent experiments.

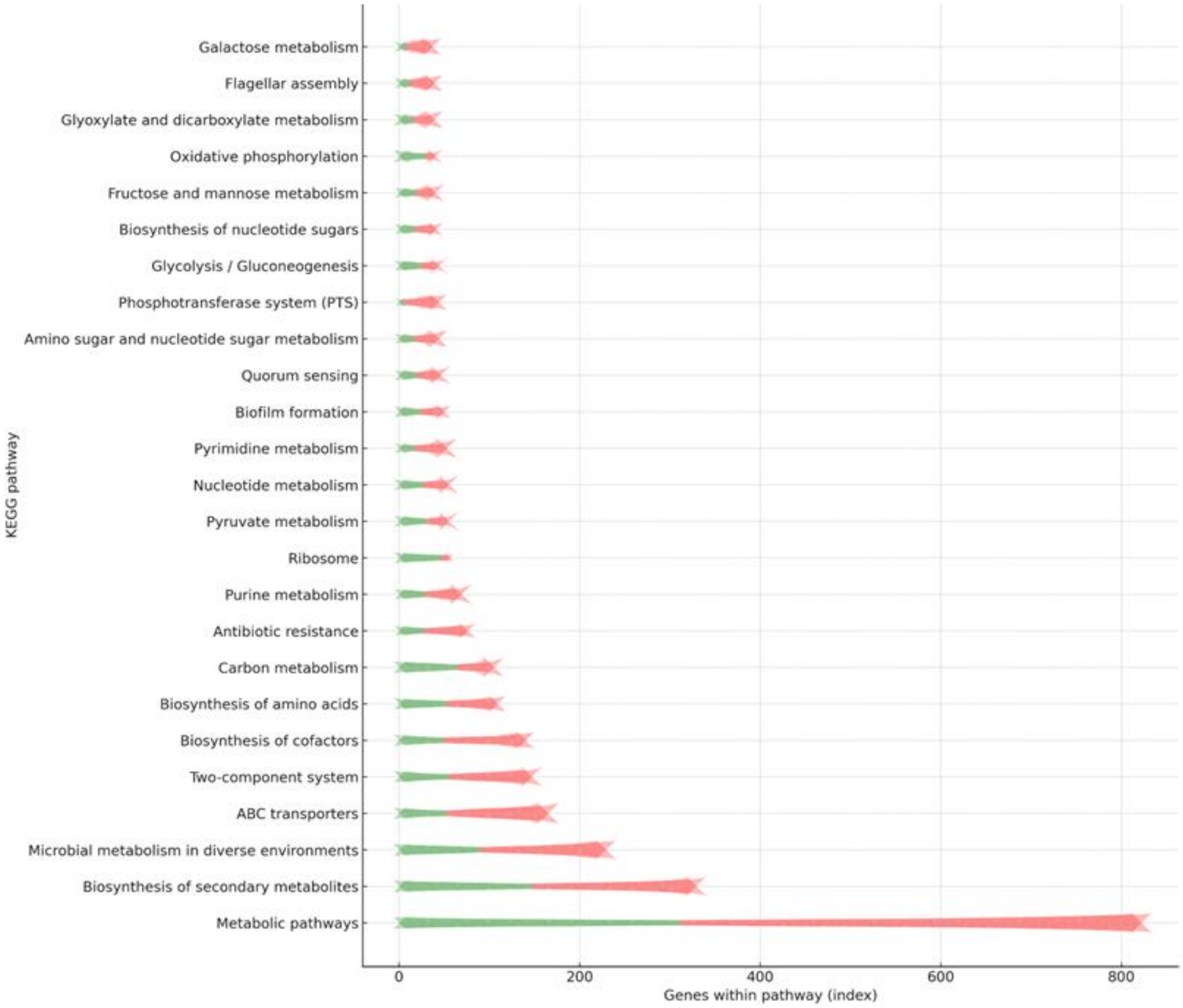

Supplementary Figure S3. KEGG pathway enrichment analysis of differentially expressed genes revealed significant upregulation of stress-response, DNA repair, and mobile-element–associated pathways (green), while biosynthetic and energy-metabolism pathways were predominantly downregulated (red).

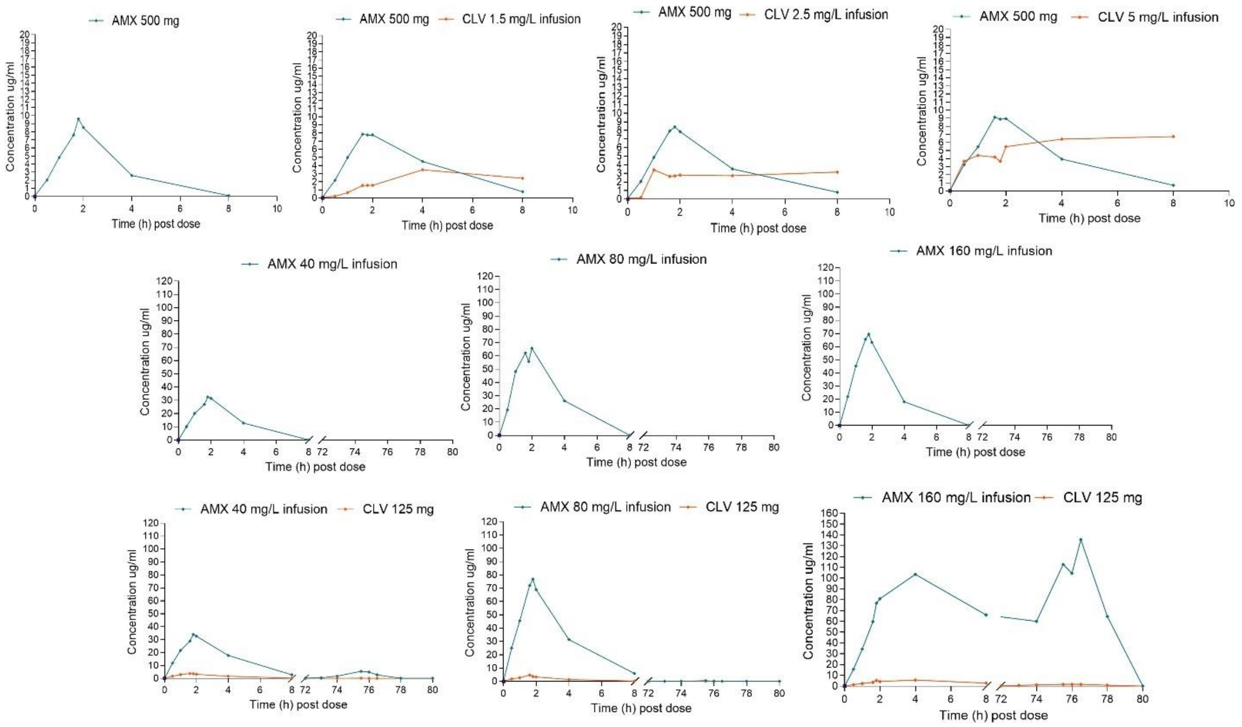

Supplementary Figure S4. Simulated human plasma pharmacokinetics of AMX and CLV in the HFIM. Concentration–time profiles for AMX and CLV measured in the hollow-fibre infection model following simulated human plasma exposures. AMX (40, 80, or 160 mg/L infusions) and CLV (1.5, 2.5 and 5 mg/L infusions along with 125 mg CLV and 500 mg AMX, respectively. Each panel shows the observed concentrations (µg/mL) of AMX (blue) and CLV (orange) over the dosing interval, beginning at time 0 h of the HFIM experiment. Data represent a single experiment (n = 1 independent HFIM run); concentrations were measured using validated LC-MS/MS methods.

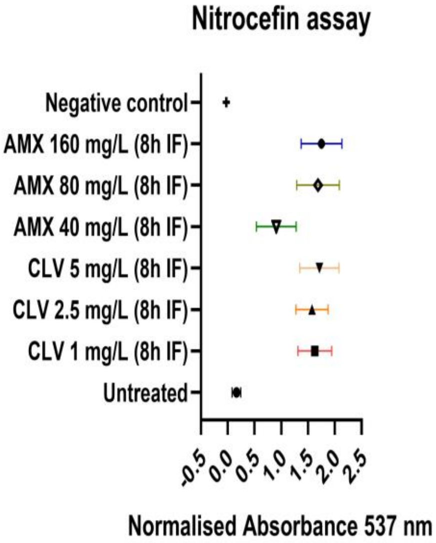

Supplementary Figure S5. Nitrocefin assay demonstrating increased β-lactamase activity in TEM-1 enriched regrowth populations. Normalised nitrocefin hydrolysis (absorbance at 537 nm) measured for *E. coli* recovered from HFIM arms exposed to AMX (40, 80 or 160 mg/L) or CLV (1, 2.5 or 5 mg/L) infusions over 8 h. Cells from drug-exposed arms exhibiting regrowth showed substantially elevated β-lactamase activity relative to untreated controls, consistent with TEM-1 overexpression and supporting the observation that enhanced β-lactam degradation limited the achievement of target AMX-CLV concentrations in the HFIM. This assay was performed in technical triplicate for each condition, Data are presented as mean±standard deviation, and are representative of three independent experiments. These data are presented for descriptive purposes to illustrate enzyme activity; no formal statistical comparisons were performed, and interpretation is based on observed trends across independent experiments.

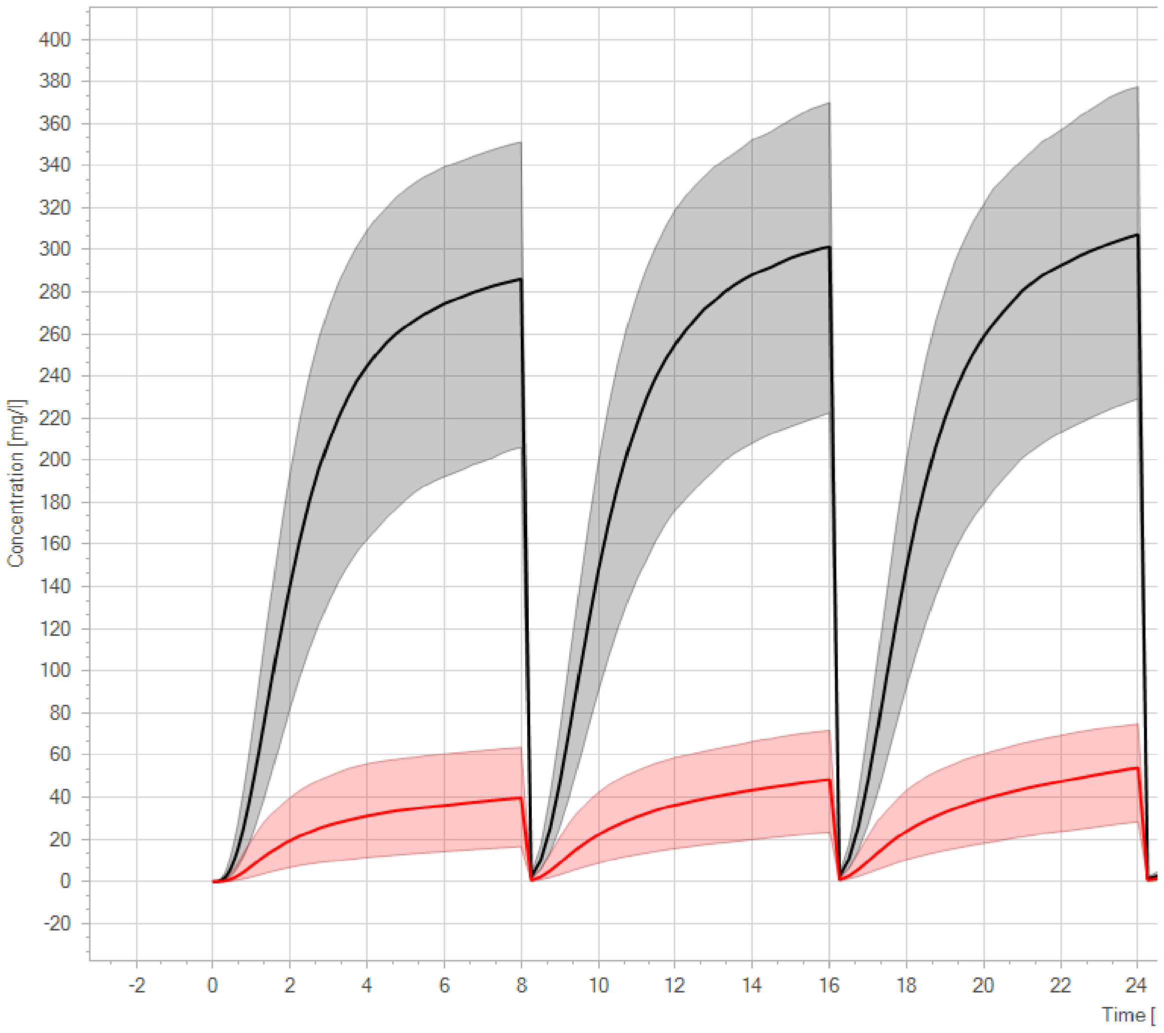

Supplementary Figure S6. Simulated urinary pharmacokinetic profiles of AMX and CLV following standard oral dosing. PBPK simulations of urinary concentrations of AMX (grey) and CLV (red) following standard oral dosing regimens (500/125 mg every 8 h). Lines indicate median predicted concentrations, and shaded regions represent inter-individual variability. Simulations assumed a urinary flow rate consistent with an 8-hour micturition frequency and Cmax values of 290 mg/L for AMX and 40 mg/L for CLV, reflecting their predominantly renal elimination. These profiles demonstrate that AMX-CLV concentrations in urine remain above therapeutic thresholds for extended periods, supporting sustained bacterial clearance and suppression of resistance emergence in the urinary tract. Data represent model simulations; n is not applicable.

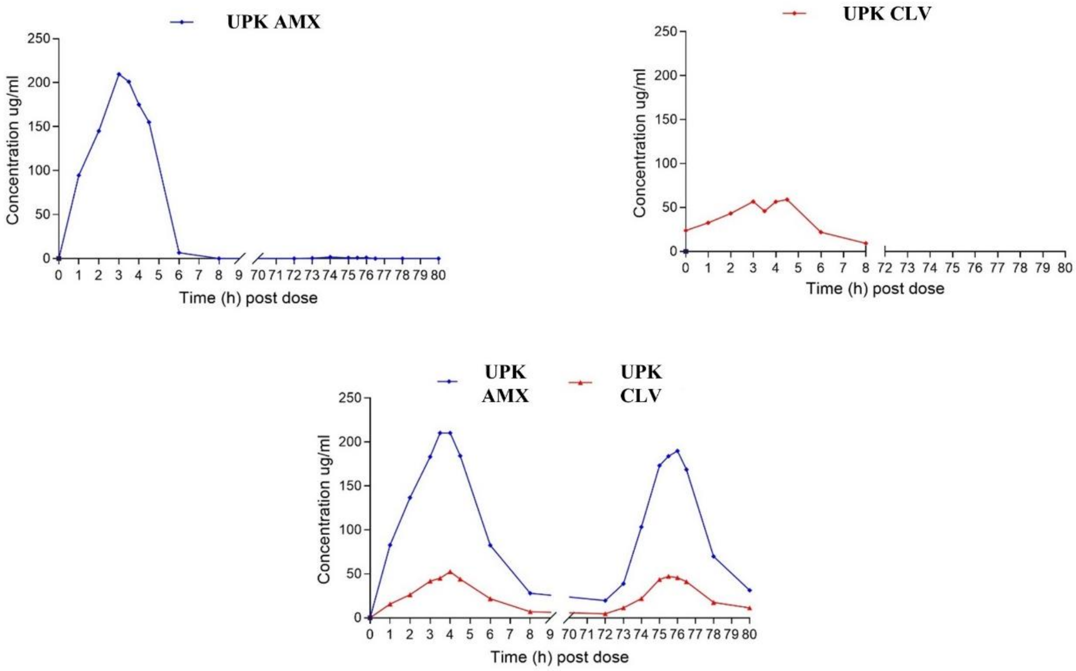

Supplementary Figure S7. Pharmacokinetics of urinary concentration-time profiles of AMX and CLV. Urinary pharmacokinetic (UPK) profiles of AMX and CLV following oral AMX-CLV dose, showing predicted drug concentrations (µg/mL) over an q8h dosing interval. Panels display the individual urinary concentration-time curves for AMX (blue) and CLV (red), as well as combined profiles where both drugs were administered together. Data represent a single experiment (n = 1 independent HFIM run); concentrations were measured using validated LC-MS/MS methods.

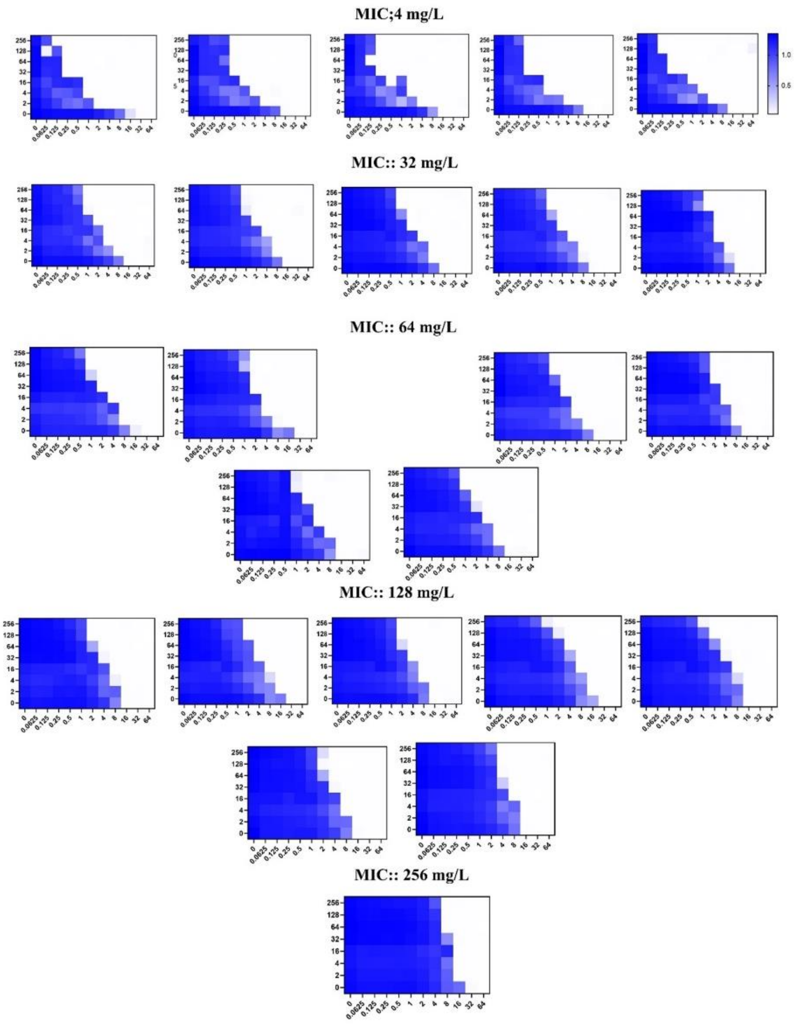

Supplementary Figure S8. Checkerboard analysis of AMX-CLV interactions across resistant *E. coli* isolates. Checkerboard assays were performed on AMX-CLV *E. coli* B50 isolates with AMX-CLV MICs ranging from 4 to 256 mg/L. Each panel shows a two-dimensional AMX-CLV concentration matrix, with growth inhibition quantified by optical density at 600 nm (OD_{600}) following overnight incubation. Heatmaps represent normalised OD_{600} values, with darker blue indicating greater inhibition and white denoting uninhibited growth (colour scale bar shown to the right). At moderate CLV concentrations, clearance depended on synergistic interactions between the β-lactam and β-lactamase inhibitor, whereas at very high CLV concentrations, direct antibacterial activity of CLV was observed. These data reveal two distinct clearance mechanisms and confirm that CLV can exert intrinsic antibacterial effects at high urinary exposures.

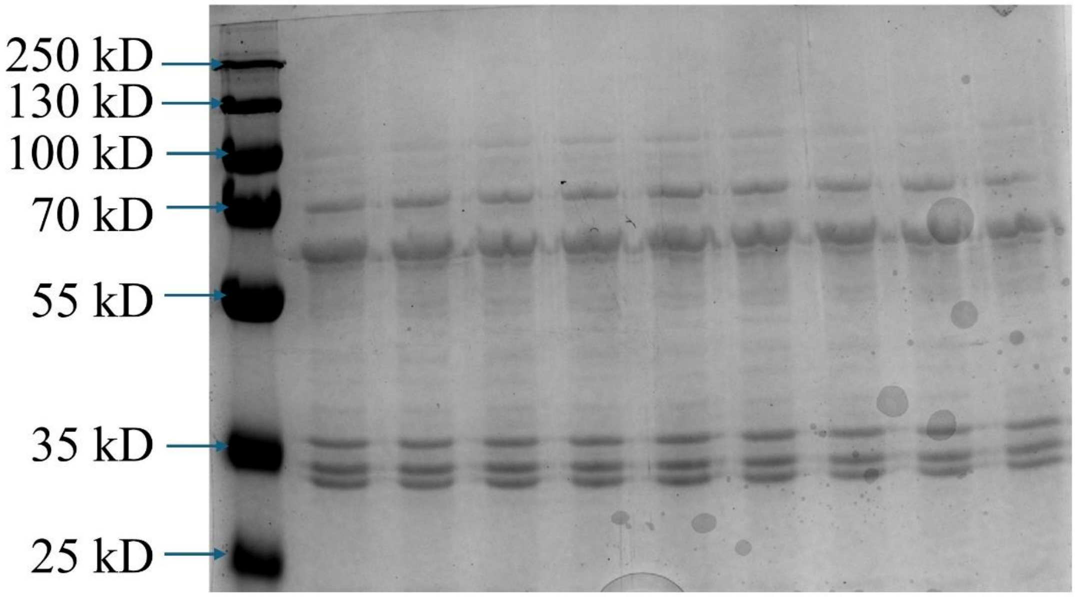

Supplementary Figure S9. Coomassie-stained SDS-PAGE confirming equal protein loading for bocillin-FL PBP labelling assays. Coomassie-stained SDS–PAGE gel of membrane preparations used for bocillin-FL competition assays performed on AMX-CLV resistant *E. coli* isolates. Molecular mass markers (kDa) are indicated above each lane. Comparable band intensities across samples confirm equivalent total protein loading, validating the interpretation of the bocillin-FL binding experiments shown in Fig. 7C.

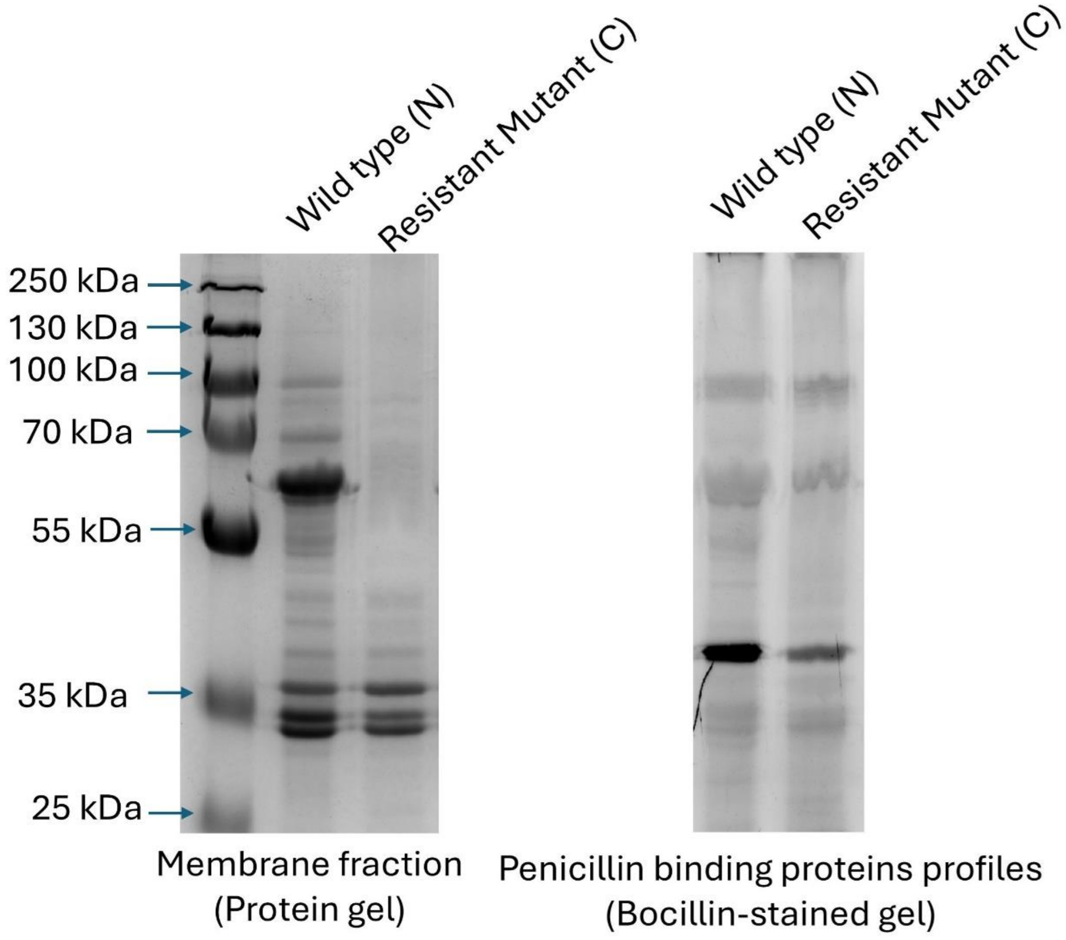

Supplementary Figure S10. Membrane protein profiles and bocillin-FL penicillin-binding protein (PBP) labelling in wild-type and CLV-resistant *E. coli* isolates. Left panel: Coomassie-stained SDS-PAGE of membrane fractions from wild-type and CLV-resistant *E. coli*, with molecular mass markers (kDa) indicated above lanes. Comparable overall banding patterns confirm equivalent membrane protein loading across samples. **Right panel:** Bocillin-FL labelled PBP profiles from the same membrane preparations. Differences in the intensity of labelled bands between wild-type and resistant mutants indicate altered PBP binding in CLV-resistant isolates, consistent with the PBP-associated susceptibility mechanisms identified in whole-genome analyses (Fig. 7E). Results are representative of three independent experiments with similar outcomes.

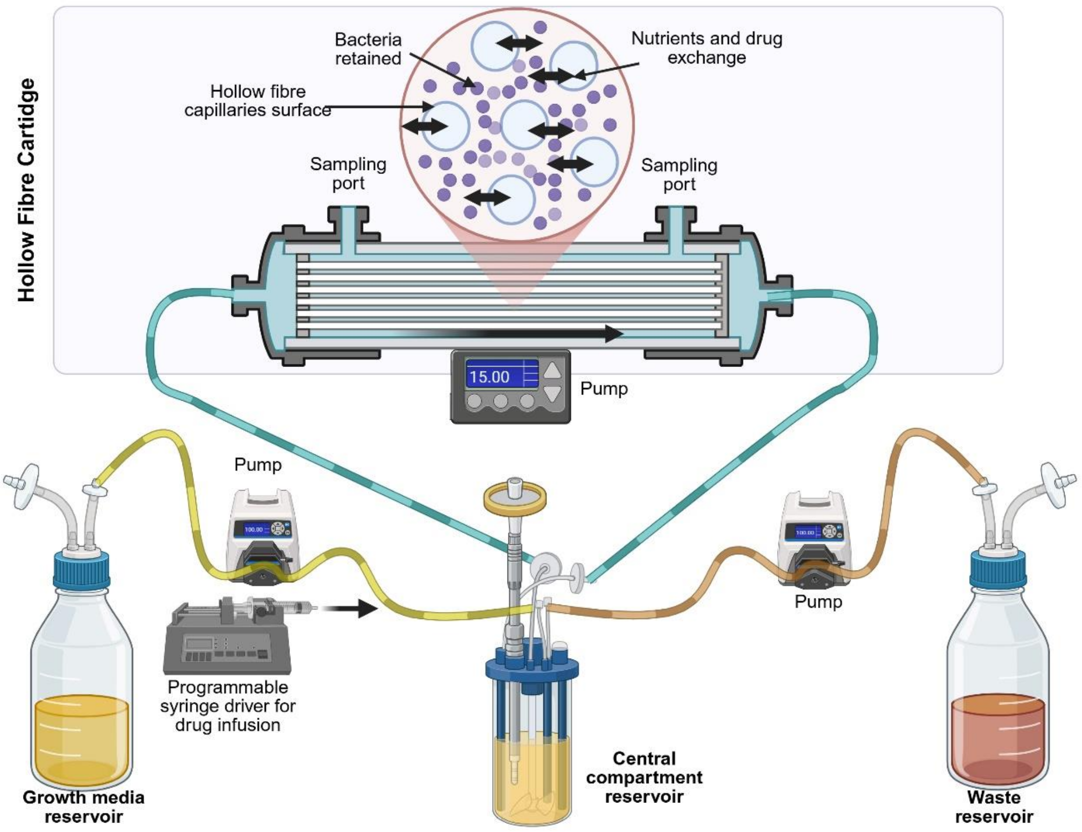

Supplementary Figure S11. HFIM Setup: Schematic representation of the HFIM system used to reproduce human drug exposure profiles. Fresh medium containing AMX and CLV is continuously infused into the central compartment by programmable syringe pumps to simulate the physiological clearance rates of both drugs. The central reservoir (200 mL) is connected to the hollow fibre cartridge, where bacteria grow on the outer surface of semi-permeable capillaries. Drug enters the extra-capillary space via diffusion, allowing bacteria to be exposed to dynamically changing concentrations over time. Waste and spent medium are removed through an elimination pump, maintaining target drug half-lives (AMX: 1.35 h; CLV: 1.15 h). Sampling ports located on both the central reservoir and the hollow fibre cartridge permit collection of pharmacokinetic and microbiological samples throughout the experiment. *Created in BioRender. Farrrington, N. (2026) https://BioRender.com/3gj3hbv*
