# Supplementary material for: Molecular pharmacodynamics of amoxicillin-clavulanic acid for urinary tract infections caused by Escherichia coli
Source: Nat Commun. 2026 Jun 13;17:7504. doi: 10.1038/s41467-026-74323-2 (PMC13408132; doi:10.1038/s41467-026-74323-2)
Supplement: Supplementary file 2 — Reporting Summary [file 41467_2026_74323_MOESM2_ESM.pdf]

nature portfolio

Corresponding author(s): William Hope

Last updated by author(s): 04-05-2026

Reporting Summary

Nature Portfolio wishes to improve the reproducibility of the work that we publish. This form provides structure for consistency and transparency in reporting. For further information on Nature Portfolio policies, see our Editorial Policies and the Editorial Policy Checklist.

Statistics

For all statistical analyses, confirm that the following items are present in the figure legend, table legend, main text, or Methods section.

n/a	Confirmed	
☐	☒	The exact sample size (*n*) for each experimental group/condition, given as a discrete number and unit of measurement
☐	☒	A statement on whether measurements were taken from distinct samples or whether the same sample was measured repeatedly
☐	☒	The statistical test(s) used AND whether they are one- or two-sided<br>*Only common tests should be described solely by name; describe more complex techniques in the Methods section.*
☒	☐	A description of all covariates tested
☐	☒	A description of any assumptions or corrections, such as tests of normality and adjustment for multiple comparisons
☐	☒	A full description of the statistical parameters including central tendency (e.g. means) or other basic estimates (e.g. regression coefficient) AND variation (e.g. standard deviation) or associated estimates of uncertainty (e.g. confidence intervals)
☐	☒	For null hypothesis testing, the test statistic (e.g. *F*, *t*, *r*) with confidence intervals, effect sizes, degrees of freedom and *P* value noted<br>*Give P values as exact values whenever suitable.*
☒	☐	For Bayesian analysis, information on the choice of priors and Markov chain Monte Carlo settings
☒	☐	For hierarchical and complex designs, identification of the appropriate level for tests and full reporting of outcomes
☒	☐	Estimates of effect sizes (e.g. Cohen's *d*, Pearson's *r*), indicating how they were calculated

Our web collection on statistics for biologists contains articles on many of the points above.

Software and code

Policy information about availability of computer code

Data collection	All software, tools, and algorithms used in this study are described in the Methods section of the manuscript, including version numbers where applicable:MassLynx Data Acquisition Software (v4.2) for LC-MS/MS data acquisitionTargetLynx Processing Software (v4.2) for bioanalysis processingFCS Express (version 7, research edition) for flow cytometry analysisADAPT 5 for pharmacokinetic/pharmacodynamic modellingPK-Sim® (Open Systems Pharmacology) for PBPK modellingBWA-MEM (v0.7.17) for sequence alignmentSAMtools (v1.10) for alignment processingPicard Tools (v2.23.3) for duplicate markingGATK (v4.2) for variant callingSnpEff (v4.2) for variant annotationSPAdes (v3.15.4) for genome assemblyBakta (v1.8) for genome annotationRGI (v6.0.0, CARD database) for AMR detectionISEScan (v1.7.2.3), ISfinder, MobileElementFinder (v1.0.3) for mobile element detectionHISAT2 (v2.2.1) for RNA-seq alignmentHTSeq (v2.0) for read countingedgeR and DESeq2 (v1.34.0) for differential expression analysisHifiasm (v0.19.5) for long-read assemblyCD-HIT (v4.8.1) for clusteringMASH (v2.3) for plasmid comparisonminimap2 (v2.26) and pbsv for structural variant detection
Data analysis	The custom code used to analyse blaTEM-1 genetic context diversity is publicly available at GitHub (https://github.com/agerada/tem-1-bootstrap). doi:10.5281/zenodo.19712613

For manuscripts utilizing custom algorithms or software that are central to the research but not yet described in published literature, software must be made available to editors and reviewers. We strongly encourage code deposition in a community repository (e.g. GitHub). See the Nature Portfolio guidelines for submitting code & software for further information.

Data

Policy information about availability of data

All manuscripts must include a data availability statement. This statement should provide the following information, where applicable:

- Accession codes, unique identifiers, or web links for publicly available datasets
- A description of any restrictions on data availability
- For clinical datasets or third party data, please ensure that the statement adheres to our policy

Sequencing data generated in this study have been deposited in the European Nucleotide Archive (ENA) under the following accession codes PRJNA1378964:[https://www.ncbi.nlm.nih.gov/bioproject/PRJNA1378964];PRJEB107892:[https://www.ebi.ac.uk/ena/browser/view/PRJEB107892]

Research involving human participants, their data, or biological material

Policy information about studies with human participants or human data. See also policy information about sex, gender (identity/presentation), and sexual orientation and race, ethnicity and racism.

Reporting on sex and gender	NA
Reporting on race, ethnicity, or other socially relevant groupings	NA
Population characteristics	NA
Recruitment	NA
Ethics oversight	NA

Note that full information on the approval of the study protocol must also be provided in the manuscript.

Field-specific reporting

Please select the one below that is the best fit for your research. If you are not sure, read the appropriate sections before making your selection.

[x] Life sciences [ ] Behavioural & social sciences [ ] Ecological, evolutionary & environmental sciences

For a reference copy of the document with all sections, see nature.com/documents/nr-reporting-summary-flat.pdf

Life sciences study design

All studies must disclose on these points even when the disclosure is negative.

Sample size	Sample sizes were determined based on established experimental standards in microbiology, pharmacodynamics, and sequencing studies, as well as consistency with prior HFIM and transcriptomic studies. Where appropriate, multiple biological replicates (typically n = 3) and technical replicates were used to ensure reproducibility and statistical robustness. No formal statistical power calculations were performed; however, the chosen sample sizes are consistent with prior literature and sufficient to detect biologically meaningful differences in bacterial growth, gene expression, and resistance phenotypes.
Data exclusions	No data was excluded from the analyses.
Replication	All the reported experiments were reproducible. Data reproducibility was confirmed by independent experiments. In vitro data used at least three biological replicates and three technical replicates in at least three independent experiments (unless stated otherwise in the manuscript).
Randomization	Samples were allocated into experimental groups based on predefined treatment conditions (e.g., antibiotic exposure regimens). Randomisation was not applicable as experiments involved controlled in vitro systems with identical starting bacterial populations. All relevant variables (growth conditions, inoculum size, media, and incubation parameters) were standardised across groups to minimise confounding effects.
Blinding	Blinding was not possible because experimental conditions such as drug concentrations, HFIM arms, sequencing groups were operationally distinct and required awareness during handling.

Reporting for specific materials, systems and methods

We require information from authors about some types of materials, experimental systems and methods used in many studies. Here, indicate whether each material, system or method listed is relevant to your study. If you are not sure if a list item applies to your research, read the appropriate section before selecting a response.

Materials & experimental systems

n/a	Involved in the study	
	x	Antibodies
x		Eukaryotic cell lines
x		Palaeontology and archaeology
x		Animals and other organisms
x		Clinical data
x		Dual use research of concern
x		Plants

Methods

n/a	Involved in the study	
x		ChIP-seq
	x	Flow cytometry
x		MRI-based neuroimaging

Antibodies

Antibodies used	All antibodies used in this study were commercially sourced and are detailed as follows: anti-mouse TEM-1 antibody (Santa Cruz Biotechnology, Cat no. sc-66062, 1:10,000), goat anti-mouse FITC-conjugated secondary antibody (Invitrogen, Cat no. 31547, 1:1,000), HRP-conjugated goat anti-mouse IgG (Abcam, Cat no. ab6728, 1:10,000), and GAPDH loading control antibody (Invitrogen, Cat no. MA5-15738).
Validation	Reguzova A, Haug V, Metz C, Müller M, Fandrich M, Dulovic A, Amann R. Heterologous prime-boost vaccination with VLA2001 and an ORFV-based vector enhances spike- and nucleocapsid-specific immunity in mice. Front Immunol. 2025 Sep 18;16:1675859. doi: 10.3389/fimmu.2025.1675859. PMID: 41050708; PMCID: PMC12488559.<br>Peng Y, Fujimura A, Asami J, Zhang Z, Shimizu T, Ohto U. Structural insights into Wnt/β-catenin signaling regulation by LGR4, R-spondin, and ZNRF3. Nat Commun. 2025 Oct 1;16(1):8337. doi: 10.1038/s41467-025-64129-z. PMID: 41034211; PMCID: PMC12488874.<br>Molecular analysis of beta-lactamase structure and function. | Majiduddin, FK., et al. 2002. Int J Med Microbiol. 292: 127-37. PMID: 12195735<br>Extended spectrum beta-lactamase producing organisms at the University Hospital of the West Indies. | Nicholson, AM., et al. 2004. West Indian Med J. 53: 104-8. PMID: 15199721<br>Biomimetic hydrolysis of penicillin G catalyzed by dinuclear zinc(II) complexes: structure-activity correlations in beta-lactamase model systems. | Bauer-Siebenlist, B., et al. 2005. Chemistry. 11: 5343-52. PMID: 16003817<br>Beta-lactamase of Bacillus licheniformis 749/C. Refinement at 2 A resolution and analysis of hydration. | Knox, JR. and Moews, PC. 1991. J Mol Biol. 220: 435-55. PMID: 1856867<br>Beta-Lactam-beta-lactamase-inhibitor combinations are active in experimental endocarditis caused by beta-lactamase-producing oxacillin-resistant staphylococci. | Hirano, L. and Bayer, AS. 1991. Antimicrob Agents Chemother. 35: 685-90. PMID: 2069374<br>The phototrophic bacterium Rhodopseudomonas capsulata sp108 encodes an indigenous class A beta-lactamase. | Campbell, JI., et al. 1989. Biochem J. 260: 803-12. PMID: 2788410<br>The active-site-serine penicillin-recognizing enzymes as members of the Streptomyces R61 DD-peptidase family. | Joris, B., et al. 1988. Biochem J. 250: 313-24. PMID: 3128280<br>Beta-lactamase-mediated resistance and opportunities for its control. | Livermore, DM. 1998. J Antimicrob Chemother. 41 Suppl D: 25-41. PMID: 9688449<br>Fan Z, Liu Y, Lin X, Zhang J, Chen J, Yi S, Hu C, Liu X, Guo C, Xu C, Chen X, Tian X, Liang X, Liu Y, Hu L, Huang S, Guo L, Zhu W, Hu J, Yan G, Lin Y, Cai J, Liang J. Suppression of multiple mouse models of refractory malignancies by reprogramming IL-18 ligand-receptor interaction. Nat Commun. 2025 Jul 3;16(1):6136. doi: 10.1038/s41467-025-61439-0. PMID: 40610476; PMCID: PMC12229637.

Plants

Seed stocks	NA
Novel plant genotypes	NA
Authentication	NA

Flow Cytometry

Plots

Confirm that:

- [x] The axis labels state the marker and fluorochrome used (e.g. CD4-FITC).
- [x] The axis scales are clearly visible. Include numbers along axes only for bottom left plot of group (a 'group' is an analysis of identical markers).
- [x] All plots are contour plots with outliers or pseudocolor plots.
- [x] A numerical value for number of cells or percentage (with statistics) is provided.

Methodology

Sample preparation	For flow cytometry bacterial cells were resuspended in 1% paraformaldehyde in PBS (pH 7.4) at a concentration of 10^6 cells/ml and incubated on ice for 20 min. The cells were washed with PBS three times, then resuspended in 70% ice-cold ethanol and allowed to stand on ice for 30 min. For staining, the cell pellet was resuspended in 1 ml wash buffer. After two washes, the pellet was incubated in 50 µl of TUNEL reaction mixture containing FITC-dUTP and deoxynucleotidyl transferase at 37°C in the dark for 1 h. After incubation, cells were washed twice with rinse buffer and incubated with 5 mg/ml BSA. Samples were washed and then resuspended in PBS for fluorescence-activated cell sorting (FACS) analysis.For cell sorting, overnight culture of E. coli BL50 was treated with 0.1 mg/ml of lysozyme at 37°C for 1 hour, followed by three washes with 20% glycerol: PBS solution. Cells were incubated with 2% BSA for 20 minutes, then incubated with 1:10000 anti-mouse TEM-1 antibody (Santa Cruz Biotechnology, Cat no: sc-66062). After three washes with 20% glycerol in PBS, the cells were incubated with a 1:1000 secondary anti-mouse goat FITC-conjugated antibody (Fisher Scientific, UK). The cell suspension was passed through the ARES III Cell Sorter instrument.
Instrument	Cytomaster flow cytometer (BD Biosciences), ARES III Cell Sorter instrument
Software	FCS express 7 research edition
Cell population abundance	Bacterial growth were performed aseptically
Gating strategy	Flow cytometry data were analysed by first gating the main bacterial population using FSC and SSC to exclude small debris. A FSC-Area vs FSC-Height gate was then applied to remove doublets. For TUNEL assays, FITC fluorescence (488-nm excitation, 525/15-nm filter) was used to quantify DNA damage; positive and negative populations were defined using unstained controls prepared in parallel. For TEM-1 expression experiments, antibody-labelled spheroplasts were gated on FSC/SSC, and FITC-positive and FITC-low subpopulations were separated based on fluorescence intensity distributions. These gates were applied consistently across all samples, and representative gating plots are provided in Supplementary Information.

- [ ] Tick this box to confirm that a figure exemplifying the gating strategy is provided in the Supplementary Information.
