# Supplementary material for: Molecular pharmacodynamics of amoxicillin-clavulanic acid for urinary tract infections caused by Escherichia coli
Source: Nat Commun. 2026 Jun 13;17:7504. doi: 10.1038/s41467-026-74323-2 (PMC13408132; doi:10.1038/s41467-026-74323-2)
Supplement: Supplementary file 3 — Transparent Peer Review file [file 41467_2026_74323_MOESM3_ESM.pdf]

nature portfolio

Peer Review File

Molecular pharmacodynamics of amoxicillin-clavulanic acid against Escherichia coli

Corresponding Author: Professor William Hope

This file contains all reviewer reports in order by version, followed by all author rebuttals in order by version.

Version 0:

Reviewer comments:

Reviewer #1

(Remarks to the Author)
This very well written manuscript presents a highly interesting and impactful study. The work provides an exceptionally comprehensive analysis of amoxicillin/clavulanic acid (AMX-CLV) pharmacology against urinary tract infections by Escherichia coli. This is paired with multiple omics techniques, flow cytometry (with sorting) and molecular analyses, and receptor binding studies, which provide a mechanistic assessment and interpretation of the important and clinically relevant findings presented.

AMX-CLV is an abundantly used antibiotic combination and E. coli is the most prevalent bacterial pathogen causing such infections, highlighting significance. The manuscript shows the simulation of plasma and urinary tract infection model concentration time profiles of AMX-CLV, which is a novel step forward, as most hollow fiber infection model studies only simulate plasma concentrations. These PK simulations were done at a state-of-the-art level. It was very interesting to see the divergent treatment outcomes between plasma and urine concentration profiles, and the manuscript assesses these both empirically and mechanistically via a series of assays and approaches.

Likewise, the IS-mediated remodeling of blaTEM-1 transposition units is novel and important, and an impactful addition to the field. The finding of an increasing gene copy number leading to beta-lactamase hyperproduction is very interesting and clinically important, and significantly enhances our understanding of this potential reason for treatment failure. Flow cytometry analysis with cell sorting based on antibody-labeling add key mechanistic insights.

The drug concentration profiles of both AMX and CLV were systematically and independently varied in this dynamic in vitro infection model, which provided additional insights that cannot be obtained in animals and humans. And the simulated PK profiles were clinically relevant.

Overall, this integrated study is extremely interesting and moves translational antimicrobial pharmacology forward, both at the mechanistic and clinical side.

Main comments:
1. Supplement Figure 1: It seems that the AMX concentrations from 72-80h were slightly lower than those during the first 8 h. The authors should briefly comment that this may have been caused by some beta-lactamase activity due to a large number of regrowing bacteria on days 2-3. The difference is not much and appears to still be in the clinical range of AMX concentrations to be expected. The authors addressed this in lines 164-167, but it would be better to provide a pointer when citing Figure S1.

2. It would be interesting, if the authors compared their heteroresistance findings about beta-lactamase copy numbers to other hetereo-resistance studies such as that by Band and Weiss (PLoS Biol 2021; 19:e3001346, PMID: 34283833).

3. The authors should briefly comment / provide a perspective on the effect of the immune system. The present manuscript shows extensive mechanistic insights which could not be created in a patient or in an animals by simulating the PK of each drug separately. Therefore, the HFIM model is highly valuable. However, the HFIM system lacks physiological aspects, including the immune system. This is not a limitation of the study, but it would widen the readers perspective.

Minor comments:
4. Line 39: Gaining mechanistic insights is undoubtedly helpful. The authors should briefly explain how they envision such novel treatment strategies. I.e. it is not clear if this is within the context of new antibiotics or by a novel use case of AMX-CLV.

5. Line 46: The word "escape" is slightly unclear, since the sentence already mentions expansion of pre-existing resistant subpopulations. Maybe "treatment failure" would be better (or just delete the last words).

6. Line 58: The authors should add a parenthesis to indicate the CLV concentration used in the combination MIC testing (e.g. 2.5 or 4 mg/L).

7. Line 73: The authors should briefly explain / justify (e.g. in the methods) the CLV concentration of 2.5 mg/L used here. This reviewer would have expected 4 mg/L as a standard CLV concentration used during susceptibility testing. The authors should also clarify whether the 32-128 mg/L is for AMX with CLV (not for AMX alone).

8. Line 161: The authors should briefly mention of they refer to oral or IV treatment for clinically relevant plasma concentrations. 160 mg/L after oral treatment with amoxicillin is above the clinical range, as indicated by the authors' text. Adding a PK reference for oral AMX-CLV would be helpful.

9. Line 224: The authors should add a reference for the observed drug conc. in urine in patients.

10. Line 235: Inhibition of PBPs by CLV as a direct killing mechanism is plausible. The authors should provide references on PBP binding by CLV (these two suggested references are on P. aerugiosa Montaner M et al. J Antimicrob Chemother 2026; 81; Ridriguez-Tebas FEMS Microbiol Lett 14, 1982, 295-8). There may be better references available for E. coli.

11. Line 354: The authors should briefly mention the HFIM cartridge material. The reader could look this up based on the catalog number, but it would be better to provide at least the material and approximate volume of the cartridge in the text.

12. References 20 and 22 appear to be duplicates.

Reviewer #2

(Remarks to the Author)
This paper addresses an important topic, ie, why amoxicillin/clavulanate is only effective for urinary tract infections. More generally, it provides insights into how antibiotic pharmacokinetics results in different selection environments for the emergence (or not) of drug resistant bacteria. The results indicate that at plasma concentrations of AMX/CLV preexisting resistant bacteria due to gene expansion of beta-lactamase leads to resistance while the urinary concentrations reach a level where these bacteria cannot survive. This provides a rationale for the clinical limitations. The results presented support the conclusions. Overall, the paper is well-written and concise. Specific comments are listed below:

1. lines 140-141. Authors state, "Notably, the convergence of oxidative-stress-associated mutations with transcriptional upregulation of IS-family transposases suggests an environment of elevated genomic plasticity." There are many mutations in many different genes and pathways. The statement is quite broad. Can this conclusion be based on a quantitative comparison or statistical analysis?
2. Table S1. Please define 'EC-5'.
3. Table S1 states 30 strains in table. There are 27 in the table.
4. Lines 63-64 states "carried different beta-lactamases". Perhaps change the wording since 2 of 3 contain the same beta-lactamase.
5. Fig S1. Was dosing every 8 hours or only at 0, 8, 72 hrs. Not clear in figure.
6. Fig. 1. Was dosing every 8 hours or only at 0?
7. Fig. 2. There is no description of panel C in the legend.
8. Fig. 3A. Mostly curiosity but what genes are at the highest two expression fold changes in the upper right of the plot? Suggest labels for these.
9. Fig. S7. All of the panels are labeled with CLV. Is this correct? Lagend says blue is AMX. Also, are the panels data or simulations? The legend says simulation.
10. Line 267, typo- "The emergence of resistance emergence.."

Reviewer #3

(Remarks to the Author)
The manuscript from Dubey et al. explores why amoxicillin–clavulanic acid is truly effective only in E. coli urinary tract infections. The authors compared a hollow fibre model mirroring the conditions of human plasma or urine drug levels, and showed that plasma like exposures rapidly selected for pre existing resistant subpopulations, whereas urine like exposures produced sustained killing without resistance. Genomics and transcriptomics revealed that plasma exposure enriches lineages carrying oxidative stress associated mutations triggering the SOS response, in turn driving insertion sequences mediated amplification of blaTEM 1, resulting in β lactamase hyperproduction and treatment failure. On the other hand, high urinary clavulanic acid concentrations induced antibacterial activity that eliminates resistant subpopulations. Taken together,

the research emphasises what is becoming a growing theme in AMR testing – that the microenvironment can strongly influence antimicrobial efficacy outcomes. In this case, it demonstrates that local pharmacokinetics strongly influence evolutionary responses to β lactam/β lactamase inhibitor therapy, explaining the site specific efficacy of AMX CLV.

This work is to my knowledge original, and is an interesting paper that adds to our knowledge about how antimicrobial efficacy is seldom black and white, and how treatment response in the clinic can't always be predicted from standard tests like MIC. It would be of interest to microbiologists (both basic researchers and clinical microbiologists) and infectious disease clinicians, especially those concerned with AMR and treatment failure. Generally the methods are sound and are described in sufficient detail, and claims supported.

I have some comments that might be considered to improve the manuscript.

Figure 1. Sorry if I missed it, but how do the authors know, at this stage, that the resistant population was only pre-existing, as opposed to some of it being selected de novo during the treatment via mutation? (It could be a mix, in theory.) This claim is made in the section head (line 66) but not necessarily backed up. If a brief explanation of how the experiments in Figure 1 prove this could be inserted, it could fend off confusion of others. If the authors don't know at this stage of the paper, the claim of pre-existence should be softened. Again unless I'm missing something, it is not until Fig 2D (line 96) where the experiment to show pre-existence is actually the case, right? The ROS quench experiment doesn't rule out other forms of ongoing mutation – but even that is reported later than the claims around Figure 1.

Figure 1. A related question, in the absence of antibiotic pressure, especially in the case of B50 and K67, is it unexpected to see so much growth of the resistant strains? Shouldn't there be a fitness cost/growth disadvantage to being capable of exerting the phenotype?

Lines 79-80. "β-lactams exert antibacterial effects beyond their primary target. Cell wall damage is known to trigger the generation of reactive oxygen species (ROS) and subsequent resistance generation." For readers who aren't AMR experts, it would be helpful to close the logical loop here and state directly that β-lactams damage the cell wall. Implied but not obvious to the uninitiated.

Fig 3A. The red labels of some of the gene names in the PCA plot are obscured by the datapoints – can these be shuffled right for better legibility?

Figure 5A. lines 178-80: "Expression increased by ~10- to >100-fold (regimen dependent), with the strongest induction in isolates exposed to AMX 500 mg/kg plus CLV 7.5-15 mg/L."
I am confused. There is no condition "AMX 500 mg/kg plus CLV 7.5-15 mg/L" on this graph. The highest Log10-fold change is for a condition labelled "AMX 500 mg/kg| CLV 25 mg/L". Perhaps an error?

In any case, I think your stats test is only comparing each treatment individually to the untreated control. You would need different comparisons to determine whether one treated condition elicited a statistically higher fold change than another – quite a lot of variability in some of those bars.

Figure 6 is called before Figure 5D – a bit confusing.

Figure 7C, lines 240-242. Would be preferable if meropenem part of the experiment was briefly explained as well in the results text and not just in the legend.

Methods: the results say the strains for initial MIC were "recovered from beads" but some description of exactly how this was done in the Methods would be useful. How bacteria are prepared prior to assays can have significant downstream effects. (PAP assay methods do this well, for example.)

Open Access This Peer Review File is licensed under a Creative Commons Attribution 4.0 International License, which permits use, sharing, adaptation, distribution and reproduction in any medium or format, as long as you give appropriate credit to the original author(s) and the source, provide a link to the Creative Commons license, and indicate if changes were made.
In cases where reviewers are anonymous, credit should be given to 'Anonymous Referee' and the source.
The images or other third party material in this Peer Review File are included in the article’s Creative Commons license, unless indicated otherwise in a credit line to the material. If material is not included in the article’s Creative Commons license and your intended use is not permitted by statutory regulation or exceeds the permitted use, you will need to obtain permission directly from the copyright holder.
To view a copy of this license, visit https://creativecommons.org/licenses/by/4.0/

REVIEWER COMMENTS

Reviewer #1 (Remarks to the Author):

Main comments:

1. Supplement Figure 1: It seems that the AMX concentrations from 72-80h were slightly lower than those during the first 8 h. The authors should briefly comment that this may have been caused by some beta-lactamase activity due to a large number of regrowing bacteria on days 2-3. The difference is not much and appears to still be in the clinical range of AMX concentrations to be expected. The authors addressed this in lines 164-167, but it would be better to provide a pointer when citing Figure S1.

We thank the reviewer for this suggestion. We have added a statement in the revised manuscript about the lower AMX concentration on the later sampling day, which is highlighted in the revised version of the manuscript (Lines 77-81).

2. It would be interesting, if the authors compared their heteroresistance findings about beta-lactamase copy numbers to other hetereo-resistance studies such as that by Band and Weiss (PLoS Biol 2021; 19:e3001346, PMID: 34283833).

We thank the reviewer for this suggestion. We have added a paragraph in the discussion section in the revised manuscript highlighting the connection between our findings and those of Band *et al.*, and we strongly believe that this inclusion has strengthened our discussion section. The addition is highlighted in the revised version of the manuscript (Line 286-297).

3. The authors should briefly comment / provide a perspective on the effect of the immune system. The present manuscript shows extensive mechanistic insights which could not be created in a patient or in an animals by simulating the PK of each drug separately. Therefore, the HFIM model is highly valuable. However, the HFIM system lacks physiological aspects, including the immune system. This is not a limitation of the study, but it would widen the readers perspective.

We thank the reviewer for this suggestion. We have added a paragraph to the Discussion in the revised manuscript that addresses the potential role of host immune responses across anatomical compartments and how these may synergise with antimicrobial activity to influence bacterial clearance. This addition is highlighted in the revised manuscript (Lines 328-335).

Minor comments:

4. Line 39: Gaining mechanistic insights is undoubtedly helpful. The authors should briefly explain how they envision such novel treatment strategies. I.e. it is not clear if this is within the context of new antibiotics or by a novel use case of AMX-CLV.

We thank the reviewer for this helpful suggestion. We have clarified the intended context of the proposed treatment strategies in the revised manuscript (Lines 39-42).

5. Line 46: The word "escape" is slightly unclear, since the sentence already mentions expansion of pre-existing resistant subpopulations. Maybe "treatment failure" would be better (or just delete the last words).

As per the reviewers' suggestion, the words "leading to escape" have been removed from the revised version of the manuscript.

6. Line 58: The authors should add a parenthesis to indicate the CLV concentration used in the combination MIC testing (e.g. 2.5 or 4 mg/L).

We thank the reviewer for this suggestion and for pointing out the error. The CLV concentration used in the combination MIC testing (2 mg/L) has now been added to the revised manuscript. (Line 60).

7. Line 73: The authors should briefly explain / justify (e.g. in the methods) the CLV concentration of 2.5 mg/L used here. This reviewer would have expected 4 mg/L as a standard CLV concentration used during susceptibility testing. The authors should also clarify whether the 32-128 mg/L is for AMX with CLV (not for AMX alone).

We thank the reviewer for this suggestion. We would like to note that standard AMX-CLV MIC susceptibility testing is performed with a fixed clavulanic acid concentration of 2 mg/L, in accordance with EUCAST guidelines, rather than 4 mg/L, which is typically used for other β-lactam/β-lactamase inhibitor combinations such as those containing avibactam. The use of 2.5 mg/L CLV in preparation of drug plates was done to closely approximate clinically relevant peak plasma concentrations simulated in the hollow-fibre infection model. We have added a statement to clarify this rationale in the revised version of the manuscript. (Lines 402-403).

8. Line 161: The authors should briefly mention of they refer to oral or IV treatment for clinically relevant plasma concentrations. 160 mg/L after oral treatment with amoxicillin is above the clinical range, as indicated by the authors' text. Adding a PK reference for oral AMX-CLV would be helpful.

We thank the reviewer for this helpful suggestion. We have added a clarifying statement to the revised manuscript to address this point (Line 163-169). Reference 25 is for oral AMX-CLV PK reference.

9. Line 224: The authors should add a reference for the observed drug conc. in urine in patients.

The references have been added in the revised manuscript (Ref. 11 and 12).

10. Line 235: Inhibition of PBPs by CLV as a direct killing mechanism is plausible. The authors should provide references on PBP binding by CLV (these two suggested references are on P. aerugiosa Montaner M et al. J Antimicrob Chemother 2026; 81; Ridriguez-Tebas FEMS Microbiol Lett 14, 1982, 295-8). There may be better references available for E. coli.

References 13 and 14 have been added relating to PBP binding by CLV

11. Line 354: The authors should briefly mention the HFIM cartridge material. The reader could look this up based on the catalog number, but it would be better to provide at least the material and approximate volume of the cartridge in the text.

Thank you for the suggestion, the details regarding the material and volume of the cartridge have been added in the revised manuscript and is highlighted for your kind attention. (Lines 393-394)

12. References 20 and 22 appear to be duplicates.

The error has been corrected.

Reviewer #2 (Remarks to the Author):

1. lines 140-141. Authors state, "Notably, the convergence of oxidative-stress-associated mutations with transcriptional upregulation of IS-family transposases suggests an environment of elevated genomic plasticity." There are many mutations in many different genes and pathways. The statement is quite broad. Can this conclusion be based on a quantitative comparison or statistical analysis?

We thank the reviewer for this important point. This statement is intended as an integrative interpretation based on the recurrent identification of mutations in oxidative stress–associated genes together with consistent transcriptional upregulation of IS-family transposases across independently evolved isolates, rather than a formal pathway-level enrichment analysis. To avoid overgeneralisation, we have revised the manuscript to clarify that this conclusion is based on repeated qualitative patterns observed across datasets rather than on a quantitative statistical comparison. (Lines 146-148)

2. Table S1. Please define 'EC-5'.

We have added the description "(EC-5: Enterobacterales Cephalosporinase, family 5)
" in table S1 legend.

3. Table S1 states 30 strains in table. There are 27 in the table.

We thank the reviewer for pointing out this error. We have amended this error in the revised supplementary file. The list of three isolates with MIC and resistance mechanisms is described in the main manuscript, and information on 27 isolates is provided in the supplementary information.

4. Lines 63-64 states "carried different beta-lactamases". Perhaps change the wording since 2 of 3 contain the same beta-lactamase.

We thank the reviewer for this suggestion, we have corrected this statement in the revised version of the manuscript. (Lines 64-66)

5. Fig S1. Was dosing every 8 hours or only at 0, 8, 72 hrs. Not clear in figure.

We thank the reviewer for this helpful comment. Drug dosing was performed every 8 hours throughout the duration of the experiment, while pharmacokinetic sampling was conducted on Day 1 and on the day prior to the end of the experiment. We have clarified this in the legend of the revised Supplementary Figure S1.

6. Fig. 1. Was dosing every 8 hours or only at 0?

Drug dosing was performed every 8 hours throughout the duration of the experiment, while pharmacokinetic sampling was conducted on Day 1 and on the day prior to the end of the experiment. We have clarified this in the legend of the revised Figure 1.

7. Fig. 2. There is no description of panel C in the legend.

We thank the reviewer for pointing out this error. We have added the description of panel C in the revised manuscript, which is highlighted for your reference.

8. Fig. 3A. Mostly curiosity but what genes are at the highest two expression fold changes in the upper right of the plot? Suggest labels for these.

The two genes with the highest expression in terms of logp-values in the upper right of Fig. 3A correspond to *gadE*, an acid resistance transcriptional activator, and a λ exonuclease family protein. Although both genes are strongly induced, their roles in amoxicillin-clavulanate resistance are not well defined. To avoid over-interpreting these findings or complicating the figure with elements not central to the study's mechanistic focus, we have chosen to retain Fig. 3A without additional gene labels. The figure is intended to illustrate global transcriptional remodelling rather than highlight specific non-causal outliers.

9. Fig. S7. All of the panels are labeled with CLV. Is this correct? Lagend says blue is AMX. Also, are the panels data or simulations? The legend says simulation.

We thank the reviewer for pointing out this error. We have corrected the figure legend S7 in the revised version.

10. Line 267, typo- "The emergence of resistance emergence.."

We thank the reviewer for pointing out this error.

Reviewer #3 (Remarks to the Author):

Figure 1. Sorry if I missed it, but how do the authors know, at this stage, that the resistant population was only pre-existing, as opposed to some of it being selected de novo during the treatment via mutation? (It could be a mix, in theory.) This claim is made in the section head (line 66) but not necessarily backed up. If a brief explanation of how the experiments in Figure 1 prove this could be inserted, it could fend off confusion of others. If the authors don't know at this stage of the paper, the claim of pre-existence should be softened. Again unless I'm missing something, it is not until Fig 2D (line 96) where the experiment to show pre-existence is actually the case, right? The ROS quench

experiment doesn't rule out other forms of ongoing mutation – but even that is reported later than the claims around Figure 1.

We thank the reviewer for this insightful comment. We agree that, at the stage of Fig. 1, our data alone cannot formally exclude a contribution from *de novo* mutation during treatment, and that the evidence for pre-existing resistance is established more directly later in the manuscript (Fig. 2D). Our intention in Fig. 1 was to describe the rapid emergence and expansion of resistant subpopulations under plasma-like AMX–CLV exposure, rather than to definitively establish their origin. To address this, we have softened the language in the section header and accompanying text associated with Fig. 1 to avoid implying that resistance is exclusively pre-existing at this stage.

Figure 1. A related question, in the absence of antibiotic pressure, especially in the case of B50 and K67, is it unexpected to see so much growth of the resistant strains? Shouldn't there be a fitness cost/growth disadvantage to being capable of exerting the phenotype?

We thank the reviewer for raising this point. We agree that most resistance mechanisms impose a fitness cost in terms of virulence and/or growth; however, this is not necessarily expected for the resistant subpopulations observed here. In our study, resistance to AMX-CLV is mediated primarily by increased TEM-1 expression rather than by target mutations, and such β-lactamase overexpression can be phenotypically heterogeneous and could only be weakly deleterious in the absence of antibiotic pressure. Also, the resistant cells detected in the untreated arms represent a minor subpopulation rather than a fixed resistant clone, and their apparent expansion reflects overall population growth rather than selective advantage. Under drug-free conditions, we did not observe evidence that resistant subpopulations outcompete sensitive cells; rather, they persist alongside them without an obvious growth penalty.

Lines 79-80. "β-lactams exert antibacterial effects beyond their primary target. Cell wall damage is known to trigger the generation of reactive oxygen species (ROS) and subsequent resistance generation." For readers who aren't AMR experts, it would be helpful to close the logical loop here and state directly that β-lactams damage the cell wall. Implied but not obvious to the uninitiated.

We have changed the statement "β-lactams exert antibacterial effects beyond their primary target" to "β-lactams inhibit penicillin-binding proteins involved in cell wall synthesis, and the resulting cell wall damage has been shown to trigger downstream stress responses, including the generation of reactive oxygen species (ROS)" in the revised version of the manuscript, which is highlighted for the reference. (Lines 85-87)

Fig 3A. The red labels of some of the gene names in the PCA plot are obscured by the datapoints – can these be shuffled right for better legibility?

We thank the reviewer for pointing out this error; the labels have been corrected in the revised Figure 3A.

Figure 5A. lines 178-80: "Expression increased by ~10- to >100-fold (regimen dependent), with the strongest induction in isolates exposed to AMX 500 mg/kg plus CLV 7.5-15 mg/L."
I am confused. There is no condition "AMX 500 mg/kg plus CLV 7.5-15 mg/L" on this graph. The

highest Log10-fold change is for a condition labelled "AMX 500 mg/kg| CLV 25 mg/L". Perhaps an error?

We thank the reviewer for pointing out this error; the labels have been corrected in the revised Figure 5A. In this figure, we compare the fold-change in *tem-1* expression with the untreated control, so we have modified the statement and highlighted it in the revised version of the manuscript (Lines 199-200).

In any case, I think your stats test is only comparing each treatment individually to the untreated control. You would need different comparisons to determine whether one treated condition elicited a statistically higher fold change than another – quite a lot of variability in some of those bars.

We agree that the tests shown in Fig. 5A compare each treatment condition independently to the untreated control and do not support formal statistical comparisons between treated conditions. We have therefore revised the text of the figure legend 5A to avoid implying that one treatment elicited a statistically greater induction than another. The revised wording clarifies that regimen-dependent differences are observed but not statistically compared across treatment arms. The variability observed across resistant isolates is consistent with heterogeneity in blaTEM-1 copy number, as shown in Fig. 5B.

Figure 6 is called before Figure 5D – a bit confusing.

We thank the reviewer for noting this inconsistency. The figure citations have been corrected so that all figures now appear in proper numerical order within the text.

Figure 7C, lines 240-242. Would be preferable if meropenem part of the experiment was briefly explained as well in the results text and not just in the legend.

We thank the reviewer for this suggestion. We have revised the Results text associated with Fig. 7C to briefly explain the inclusion of meropenem in the Bocillin FL competition assay. Meropenem is now explicitly described as a positive control for penicillin-binding protein (PBP) engagement, thereby validating the assay's specificity and interpretability. (Line 253-254)

Methods: the results say the strains for initial MIC were "recovered from beads" but some description of exactly how this was done in the Methods would be useful. How bacteria are prepared prior to assays can have significant downstream effects. (PAP assay methods do this well, for example.)

We thank the reviewer for this suggestion, we have clarify the method is the revised manuscript (Lines 347-356)
